# Targeting IRG1 reverses the immunosuppressive function of tumor-associated macrophages and enhances cancer immunotherapy

**DOI:** 10.1126/sciadv.adg0654

**Published:** 2023-04-28

**Authors:** Yu-Jia Chen, Guan-Nan Li, Xian-Jing Li, Lin-Xing Wei, Min-Jie Fu, Zhou-Li Cheng, Zhen Yang, Gui-Qi Zhu, Xu-Dong Wang, Cheng Zhang, Jin-Ye Zhang, Yi-Ping Sun, Hexige Saiyin, Jin Zhang, Wei-Ren Liu, Wen-Wei Zhu, Kun-Liang Guan, Yue Xiong, Yong Yang, Dan Ye, Lei-Lei Chen

**Affiliations:** ^1^ Shanghai Key Laboratory of Clinical Geriatric Medicine, Huadong Hospital, Fudan University; Shanghai Key Laboratory of Medical Epigenetics, International Co-laboratory of Medical Epigenetics and Metabolism (Ministry of Science and Technology); Key Laboratory of Metabolism and Molecular Medicine (Ministry of Education); Molecular and Cell Biology Lab, Institutes of Biomedical Sciences, Shanghai Medical College of Fudan University, Shanghai, China.; ^2^ State Key Laboratory of Natural Medicines, China Pharmaceutical University, Nanjing, Jiangsu, China.; ^3^ Department of Neurosurgery, Huashan Hospital, Fudan University, Shanghai, China.; ^4^ Department of Liver Surgery, Liver Cancer Institute, Zhongshan Hospital, Fudan University, Key Laboratory of Carcinogenesis and Cancer Invasion of the Ministry of Education, Shanghai, China.; ^5^ Center for Stem Cell and Regenerative Medicine, Department of Basic Medical Sciences, and Bone Marrow for Transplantation Center of the First Affiliated Hospital, Zhejiang University School of Medicine, Hangzhou 310058, China.; ^6^ State Key Laboratory of Genetic Engineering, School of Life Sciences, Fudan University, Shanghai 200433, China.; ^7^ Zhejiang Laboratory for Systems and Precision Medicine, Zhejiang University Medical Center, Hangzhou 311121, Zhejiang Province, China.; ^8^ Department of Pharmacology and Moores Cancer Center, University of California San Diego, La Jolla, CA 92037, USA.; ^9^ Cullgen Inc., 12671 High Bluff Drive, San Diego, CA 92130, USA.; ^10^ Department of General Surgery, Huashan Hospital, Fudan University, Shanghai 200040, China.

## Abstract

Immune-responsive gene 1 (IRG1) encodes aconitate decarboxylase (ACOD1) that catalyzes the production of itaconic acids (ITAs). The anti-inflammatory function of IRG1/ITA has been established in multiple pathogen models, but very little is known in cancer. Here, we show that IRG1 is expressed in tumor-associated macrophages (TAMs) in both human and mouse tumors. Mechanistically, tumor cells induce *Irg1* expression in macrophages by activating NF-κB pathway, and ITA produced by ACOD1 inhibits TET DNA dioxygenases to dampen the expression of inflammatory genes and the infiltration of CD8^+^ T cells into tumor sites. Deletion of *Irg1* in mice suppresses the growth of multiple tumor types and enhances the efficacy of anti–PD-(L)1 immunotherapy. Our study provides a proof of concept that ACOD1 is a potential target for immune-oncology drugs and *IRG1*-deficient macrophages represent a potent cell therapy strategy for cancer treatment even in pancreatic tumors that are resistant to T cell–based immunotherapy.

## Teaser

Targeting IRG1 skews tumor associated macrophages (TAMs) into anti-tumor mode and enhances responses to anti-PD-(L)1 immunotherapy.

## INTRODUCTION

Cancer immunotherapy has revolutionized cancer treatment. Exemplified by the success of immune checkpoint inhibition using antibodies against PD-1 and PD-L1 and chimeric antigen receptor-T (CAR-T) cell therapies, cancer immunotherapy has achieved remarkably effective and durable responses in a subset of patients with different cancer types. However, most patients receiving these therapies, even in combination, do not derive clinical benefit (*1*). Further development of agents, including small molecules (*2*), and strategies targeting additional immune checkpoints is critical.

Macrophages are crucial innate immune effector cells that perform a multitude of functions in response to pathogens and tissue inflammatory signals, including tumor development. Tumor-associated macrophages (TAMs) represent one of the most abundant immune cell types in the tumor stroma, and infiltration of macrophages is associated with poor prognosis and correlates with chemotherapy resistance in most cancers (*3*). In a widely used model, TAMs undergo polarization into two forms: the “classical” (or M1-polarized) form that is involved in the responses of T helper 1 (T_H_1) cells and produces proinflammatory antitumor effects, and the “alternative” (or M2-polarized) form that is involved in T_H_2-type responses and produces anti-inflammatory mediators to contribute many protumorigenic outcomes in cancer (*4*). A better understanding of the regulation of TAM polarization may identify additional immune checkpoint targets to achieve more durable antitumor immunity in broader patient populations.

The rapid proliferation of cancer cells in growing tumors results in a local change of oxygen, nutrients, and metabolites in the tumor microenvironment (TME). The alterations of TME cause a metabolic reprogramming of tumor-associated immune cells, such as T cells and macrophages (*5*). One marked metabolic change is the rapid accumulation of a previously understudied metabolite, itaconic acid (ITA), from a barely detectable low level in resting cells to 3 to 4 mM in classically activated macrophages (*6*). This is resulted from transcriptional activation of the immune-responsive gene (*IRG1*), which encodes the metabolic enzyme aconitate decarboxylase (ACOD1) that catalyzes the production of ITA (*6*, *7*). Genetic analyses of *Irg1*-deficient mice have established the anti-inflammatory function of IRG1/ITA in multiple pathogen models, including sepsis, viral infections, psoriasis, gout, ischemia/reperfusion injury, and pulmonary fibrosis (*8*–*13*). Emerging evidence suggests that IRG1 exhibits tumor-promoting properties by activating MAPK cascade in tumor cells (*14*), but very little is known about its biological function in tumor immunity in vivo.

In this study, we present evidence that tumor cells induce IRG1 expression in TAM subsets by nuclear factor κB (NF-κB) activation, underlying the cross-talk between tumor cells and macrophages. Irg1 regulates TAM polarization and promotes their immune suppressive function to potentiate tumor outgrowth. *Irg1*-deficient mice are resistant to tumor growth and have enhanced responses to anti–PD-(L)1 immunotherapy. Infusion of *Irg1*-deficient macrophages into wild-type mice inhibits syngeneic tumor growth. These results thus identify ACOD1 as a potential target for cancer immunotherapy and *IRG1*-deficient macrophages as a potent cell therapy strategy for cancer treatment.

## RESULTS

### *Irg1* deficiency inhibits tumor growth in immune-competent mice

**Fig. 1.**
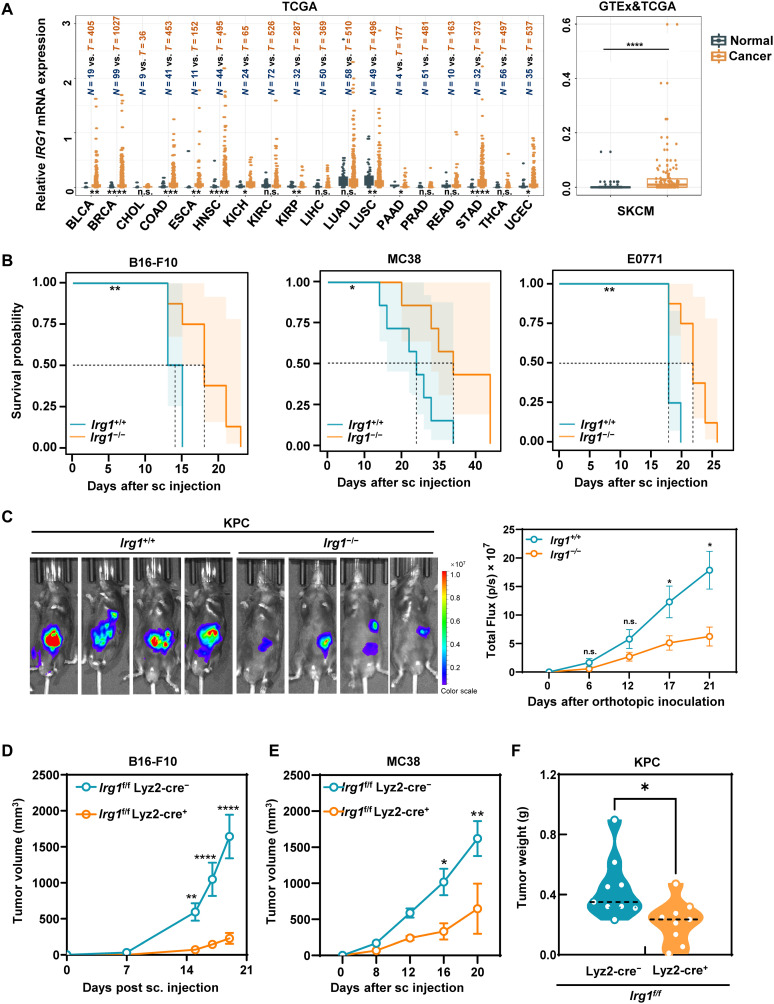
*Irg1* deficiency inhibits tumor growth in immune competent mice. (**A**) mRNA expression of *IRG1* was compared across 19 distinct solid tumors (*T*) and corresponding normal tissues (*N*) from the TCGA pan-cancer dataset. Asterisks denote statistical significance with Wilcoxon rank sum test. (**B**) B16-F10 (left), MC38 (middle), and E0771 (right) were subcutaneously (sc) injected in *Irg1*^+/+^ and *Irg1*^−/−^ mice (*n* = 7 to 8 per group) and overall survival of the mice was shown. The *P* values were calculated using log-rank (Mantel-Cox) test. (**C**) KPC pancreatic cancer cells (FC1242) overexpressing luciferase were inoculated orthotopically in the pancreas of *Irg1*^+/+^ and *Irg1*^−/−^ mice (*n* = 7). Tumor growth was observed and quantified by bioluminescent imaging. Representative live images (left) and quantified results (right) were shown, respectively. Data are means ± SEM. The *P* values were calculated by two-way analysis of variance (ANOVA). (**D** and **E**) B16-F10 (D) and MC38 (E) were subcutaneously injected in *Irg1*^f/f^ and *Irg1*^f/f^ Lyz2-cre^+^ mice (*n* = 6 to 12 per group), and tumor growth was determined by the measurement of tumor volume (cubic millimeter). Data are means ± SEM, and the *P* values were calculated by two-way ANOVA. (**F**) KPC pancreatic cancer cells (FC1242) were inoculated orthotopically in the pancreas of *Irg1*^f/f^ and *Irg1*^f/f^ Lyz2-cre^+^ mice (*n* = 9 per group). Tumors were collected at day 15 after inoculation, and tumor weight was measured. The *P* values were calculated by unpaired, two-tailed Student’s *t* test. *P < 0.05, **P < 0.01, ***P < 0.001, and ****P < 0.0001. n.s., nonsignificant.

The broad induction of *IRG1* transcription by various pathogens and the anti-inflammatory activity of ITA prompted us to explore the function of IRG1 in tumorigenesis. We first analyzed the expression of *IRG1* gene in patients from The Cancer Genome Atlas (TCGA) cohorts and found that *IRG1* mRNA expression was up-regulated in multiple types of tumors as compared to the corresponding para-carcinoma normal tissues, including BLCA (bladder urothelial carcinoma), BRCA (breast invasive carcinoma), COAD (colon adenocarcinoma), ESCA (esophageal carcinoma), HNSC (head and neck squamous cell carcinoma), KICH (kidney chromophobe), KIRP (kidney renal papillary cell carcinoma), LUSC (lung squamous cell carcinoma), PAAD (pancreatic adenocarcinoma), STAD (stomach adenocarcinoma), and UCEC (uterine corpus endometrial carcinoma) (Fig. 1A). Likewise, *IRG1* mRNA expression was up-regulated in patients with SKCM (skin cutaneous melanoma) compared with healthy skin tissues (Fig. 1A). By using the computational method EPIC (estimating the proportions of immune and cancer cells), we found that *IRG1* mRNA expression was positively correlated with the TAM fraction in a broad spectrum of human cancers (fig. S1). In accord, single-cell RNA sequencing (scRNA-seq) in samples from patients with uveal melanoma revealed that IRG1 was highly expressed in TAMs, but not other types of immune cells (fig. S2A) (*15*). In addition, this expression pattern of IRG1 was also observed in monocytes and macrophages in the TME of 12 other types of human cancers (fig. S2B) (*16*).

To decipher how IRG1 expression is induced in TAMs, we cocultured mouse bone marrow–derived macrophages (BMDMs) with different types of syngeneic tumor cells or treated BMDMs with tumor cell–conditioned medium (TCM) and found that either treatment led to *Irg1* induction and ITA accumulation (to millimolar level) in BMDMs (fig. S3, A and B). It has previously been reported that IRG1 expression is regulated by signaling pathways under inflammatory conditions, such as NF-κB (*17*), glucocorticoid receptor (GR), and Janus kinase (JAK)/signal transducer and activator of transcription (STAT) signaling pathways (*18*). We found that the effect of B16-F10-TCM on up-regulating *Irg1* expression was diminished by the treatment with pyrrolidine dithiocarbamate (an NF-κB inhibitor), but not Ruxolitinib (Ruxo, a JAK1/2 inhibitor) (fig. S3C). *Il-6* and *Il-1b* were included as two positive control genes (fig. S3C). In addition, we found that occupancy of RelA/P65 at the promoter region of *Irg1* was increased in BMDMs by the treatment of B16-F10-TCM (fig. S3D), further supporting that tumor cells induce *Irg1* expression in macrophages through NF-κB activation.

To explore the biological function of Irg1 during tumor development, we generated different syngeneic tumor models in which B16-F10 (melanoma), MC38 (colon cancer), and E0771 (breast cancer) cells were inoculated subcutaneously into *Irg1*^−/−^ and *Irg1*^+/+^ mice. We found that, in all the abovementioned tumor models, the tumor growth was significantly suppressed in *Irg1*^−/−^ mice (fig. S4A), and the survival of tumor-bearing mice was prolonged (Fig. 1B). Furthermore, pancreatic cancer cells derived from the genetically engineered mouse tumor model (LSL-KrasG12D; LSL-Trp53R172H; Pdx1-Cre mice; KPC) were implanted into the pancreas of *Irg1*^−/−^ and *Irg1*^+/+^ mice, and the KPC tumor growth was also remarkably inhibited in *Irg1*^−/−^ mice as monitored by bioluminescent imaging (Fig. 1C).

It is known that Acod1 produces ITA under inflammatory conditions, principally in cells of myeloid lineage (*19*). We found that, in the TME of B10-F10 tumors, the accumulation of ITA was readily detected in myeloid cells (CD45^+^CD11b^+^) including TAMs (F4/80^+^Cd45^+^), but not in lymphocytes (CD45^+^CD11b^−^) or nonimmune cells (CD45^−^CD11b^−^) (fig. S4, B and C). We then generated myeloid-specific *Irg1*-deficient mice (Irg1^f/f^Lyz2-cre^+^) and verified the proper deletion of *Irg1* in cells of myeloid lineage in these animals (fig. S5, A to C). Consistent with the whole-body knockout (KO), myeloid-specific KO of *Irg1* inhibited the growth of B16-F10, MC38, and KPC tumors in mice (Fig. 1, D to F, and fig. S5, D and E). Collectively, these results suggest that Irg1 in myeloid cells potentiates tumor growth in immunocompetent mice.

### *Irg1* deficiency reverses the immunosuppressive TME

**Fig. 2.**
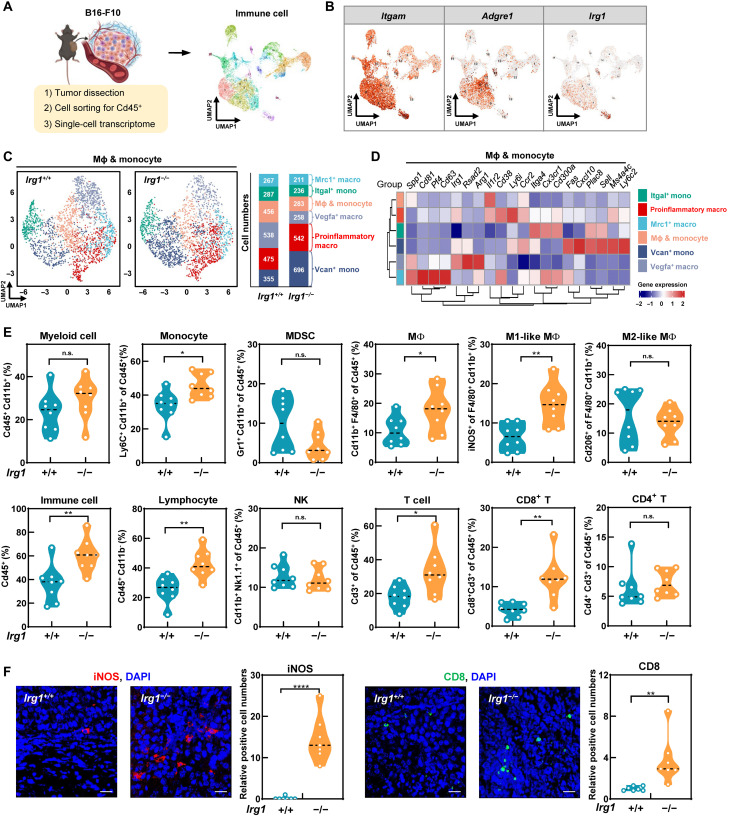
*Irg1* deficiency reverses the immunosuppressive TME. (**A**) Experimental schematics of scRNA-seq (left). Briefly, melanoma samples from *Irg1*^+/+^ or *Irg1*^−/−^ mice (*n* = 3 per group) were dissected, and Cd45^+^ immune cells were sorted by flow cytometry and subjected to transcriptome sequencing. The UMAP plot of 19 clusters is shown (right). (**B**) Expression of *Irg1* and macrophage marker genes was illustrated in the UMAP plots. (**C**) Annotated clusters of intratumoral monocytes/macrophages in *Irg1*^+/+^ or *Irg1*^−/−^ mice are shown by t-SNE plots (left). Cell numbers of each cluster are listed (right). (**D**) Normalized expression of select genes in each TAM subtype is shown by heatmap. (**E**) Flow cytometry analysis of immune cells in B16-F10 tumors from *Irg1*^+/+^ or *Irg1*^−/−^ mice (*n* = 8 per group). Tumors were harvested at 14 days after inoculation. Percentages of myeloid cells (Cd45^+^Cd11b^+^), monocytes (Cd45^+^Cd11b^+^Ly6c^+^), MDSC (Cd45^+^Cd11b^+^Gr1^+^), macrophages (Cd45^+^Cd11b^+^F4/80^+^), M1-like macrophages (Cd45^+^Cd11b^+^F4/80^+^iNos^+^), M2-like macrophages (Cd45^+^Cd11b^+^F4/80^+^Cd206^+^), lymphocytes (Cd45^+^Cd11b^−^), T cells (Cd45^+^Cd3^+^), CD8^+^ T cells (Cd45^+^Cd3^+^Cd8^+^), CD4^+^ T cells (Cd45^+^Cd3^+^Cd4^+^), and natural killer (NK) cells (Cd45^+^Cd11b^+^Nk1.1^+^) are shown by violin plot. (**F**) IF staining of iNOS^+^ and CD8^+^ cells in B16-F10 tumors from *Irg1*^+/+^ or *Irg1*^−/−^ mice. The tumors were harvested at endpoint of the survival assay as described in Fig. 1B. Scale bars, 20 μm. To quantify, cell number from seven random high-power fields (HPFs) is shown by violin plot. The *P* values were calculated by unpaired, two-tailed Student’s *t* test. *P < 0.05, **P < 0.01, and ****P < 0.0001.

To evaluate the impact of Irg1 on the TME, immune cells (Cd45^+^) were purified from B16-F10 tumors grown in *Irg1*^+/+^ and *Irg1*^−/−^ mice and then subjected to scRNA-seq (Fig. 2A and table S1). Using Uniform Manifold Approximation and Projection (UMAP) clustering analyses, 19 clusters of major immune populations were distinguished (fig. S6A), and these clusters were assigned to eight different types of immune cells, including macrophages (MФ) and monocytes, T cells, B cells, natural killer (NK) cells, plasmacytoid dendritic cells, dendritic cells, neutrophils, and limited leaked melanocytes (fig. S6, B and C). As expected, *Irg1* mRNA expression was readily detected in myeloid cells characterized by high expression of *Itgam* (Cd11b), particularly in macrophages characterized by high expression of *Adgre1* (F4/80) and neutrophils characterized by high expression of *Retnlg* or *S100a9* in the TME of B16-F10 tumors from *Irg1*^+/+^ mice (Fig. 2B and fig. S6D).

Next, we used UMAP clustering and divided the population of “MФ and monocytes” into six distinct clusters. We found that *Irg1* was mainly expressed in a few subpopulations, including Vcan^+^ monocytes, proinflammatory macrophages, and Vegfa^+^ macrophages (Fig. 2, C and D). Among these clusters, Vcan^+^ monocytes exhibited high expression of *Ly6c2*, *Cxcl10*, and *Vcan* and enriched in type I and II interferon (IFN) pathways (fig. S7, A and B), which are similar with hemopoietic system-derived classical inflammatory monocytes as previously termed (*20*). Proinflammatory macrophages exhibited high expression of major histocompatibility complex class II (MHC-II) (e.g., *H2-Aa*, *H2-Ab1*, *H2-DMb1*, and *H2-Eb1*) (fig. S7A), which are similar with the classical activated and M1-like macrophages (*21*). Vegfa^+^ macrophages exhibited high expression of *Spp1*, *Vegfa*, and *Mmp12*, and enriched in anti-inflammatory pathways, sharing the similarity with the previously termed SPP1^+^ macrophages, which have an enrichment of tumor angiogenesis and protumorigenic role (*20*). Mrc1^+^ macrophages were found to show high expression of *Mrc1*, *Retnla*, and *Gatm* (fig. S7A), sharing the similarity with alternatively activated and M2-like macrophages (*21*, *22*). According to the pseudotime-organized sequence of “differentiation/activation” events, Vegfa^+^ macrophages, together with proinflammatory macrophages and Mrc1^+^ macrophages, were differentiated from Vcan^+^ monocytes and Itgal^+^ monocytes (fig. S7C). We found that *Irg1* deficiency in mice led to the increase of Vcan^+^ monocytes and proinflammatory macrophages and a decrease of Vegfa^+^ macrophages in the TME of B16-F10 tumors (Fig. 2C), indicating that Irg1 may regulate TAM polarization during tumor development.

To validate the abovementioned observations, we carried out flow cytometric analysis and demonstrated that the populations of tumor-infiltrating monocytes (Ly6C^+^Cd11b^+^Cd45^+^) and M1-like TAMs (iNOS^+^F4/80^+^Cd11b^+^Cd45^+^) were significantly increased in the TME of B16-F10 tumors from *Irg1*^−/−^ mice compared with *Irg1*^+/+^ controls (Fig. 2E and fig. S8). No significant changes in the population of M2-like TAMs (Cd206^+^F4/80^+^Cd11b^+^Cd45^+^) and myeloid-derived suppressor cells (MDSCs) (Gr1^+^Cd11b^+^Cd45^+^) were observed in the TME of B16-F10 tumors from *Irg1*^−/−^ mice compared with *Irg1*^+/+^ controls (Fig. 2E). In addition, measurement of tumor-infiltrating lymphocytes revealed a significant increase in the population of T cells (Cd3^+^Cd45^+^), especially CD8^+^ T (Cd8^+^Cd3^+^Cd45^+^), in the TME of B16-F10 tumors from *Irg1*^−/−^ mice compared with *Irg1*^+/+^ controls (Fig. 2E). Immunofluorescent staining confirmed more iNOS^+^ or CD8^+^ cells in the TME of B16-F10 tumors growing in *Irg1*^−/−^ mice than *Irg1*^+/+^ controls (Fig. 2F). Together, these findings indicate that *Irg1* deficiency in mice is associated with reduced immunosuppressive TME and thus supports the immunosuppressive function of IRG1 during tumor development.

### *Irg1-*deficient macrophages acquire more proinflammatory features and promote antigen presentation and T cell chemotaxis

**Fig. 3.**
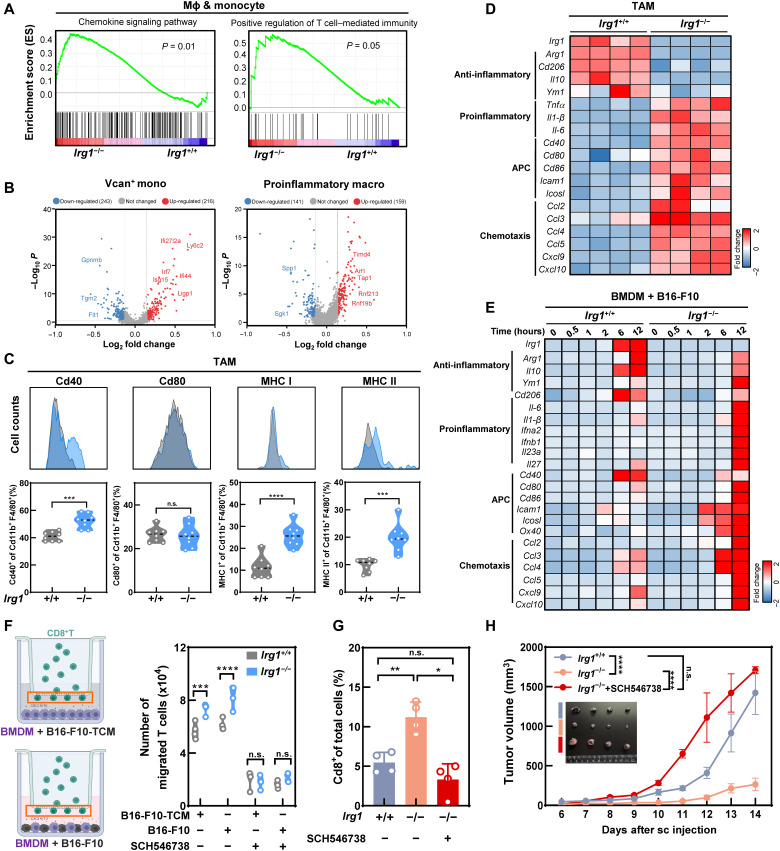
*Irg1*-deficient macrophages acquire more proinflammatory features and promote antigen presentation and T cell chemotaxis. (**A**) Enrichment score of indicated pathways in monocytes/macrophages from *Irg1*^+/+^ or *Irg1*^−/−^ mice, according to scRNA-seq data in Fig. 2A. (**B**) Volcano plots of log_2_ fold change and log_10_ adjusted *P* value of differentially expressed genes in Vcan^+^ monocytes or Vegfa^+^ macrophages from *Irg1*^+/+^ and *Irg1*^−/−^ mice. (**C**) Antigen presentation machinery in TAMs from the TME of B16-F10 tumors in *Irg1*^+/+^ and *Irg1*^−/−^ mice (*n* = 8 per group), as determined by flow cytometry with indicated antibodies. The *P* values were calculated by an unpaired, two-tailed Student’s *t* test. (**D**) Gene expression heatmap in TAMs of B16-F10 tumors from *Irg1*^+/+^ and *Irg1*^−/−^ mice (*n* = 4 per group). (**E**) Gene expression heatmap in BMDMs cocultured with B16-F10 for indicated time points. (**F**) BMDMs were cocultured with B16-F10 or stimulated with B16-F10-TCM in the lower chambers of a 5-μm Transwell plate. CD8^+^ T cells that migrated into the lower chambers were collected and counted. Data shown are from four independent experiments. The *P* values were calculated by two-way ANOVA. (**G** and **H**) *Irg1*^+/+^ and *Irg1*^−/−^ mice were subcutaneously injected with B16-F10 cells and treated with PBS or SCH546738 (*n* = 4 per group). The percentages of CD8^+^ T cells (Cd3^+^Cd8^+^) were determined by flow cytometry (G), and B16-F10 tumor growth (H) was determined by the measurement of tumor volume (cubic millimeter). The *P* values were calculated by one-way ANOVA (G) and two-way ANOVA (H). *P < 0.05, **P < 0.01, ***P < 0.001, and ****P < 0.0001.

We recently reported that ITA is structurally similar to α-ketoglutaric acid (α-KG) and binds to and inhibits α-KG–dependent TET (ten-eleven translocation) DNA demethylases, thereby down-regulating NF-κB and STAT target genes to dampen the inflammatory responses (*9*). The expression of IRG1 in macrophages in both human and mouse tumors and the association of IRG1 with immunosuppressive TME led us to further characterize the effect of IRG1 on the transcriptomic landscape in TAMs. To this end, gene set enrichment analysis (GSEA) and single-sample GSEA were conducted to calculate gene signature–based scores. We found that intratumoral monocytes/macrophages in melanoma from *Irg1*^−/−^ mice exhibited higher gene signature scores of cytokine production, antigen processing and presentation, chemokine signaling pathway, and positive regulation of T cell migration as compared with those cells from *Irg1*^+/+^ controls (Fig. 3A and fig. S9A). Among the population of MФ and monocytes, Vcan^+^ monocytes exhibited up-regulation of IFN-stimulated genes (e.g., *Irf7*, *Isg15*, *Ifi44*, and *Ifi27l2a*) in the TME of B16-F10 tumors from *Irg1*^−/−^ mice compared with *Irg1*^+/+^ controls, and genes associated with antigen presentation and T cell migration (e.g., *Aif1*, *Timd4*, and *Tap1*) were also up-regulated in proinflammatory macrophages from tumor-bearing *Irg1*^−/−^ mice (Fig. 3B). In accord, flow cytometry analysis illustrated that TAMs from tumor-bearing *Irg1*^−/−^ mice manifested enhanced immunogenic antigen presentation, as evidenced by higher expression of cell surface markers, such as MHC-I, MHC-II, or Cd40 (Fig. 3C).

In agreement with the aforementioned observation (Fig. 2E), transcriptional analysis by quantitative reverse transcription polymerase chain reaction (qRT-PCR) showed that TAMs from tumors grown in *Irg1*^−/−^ mice exhibited more of proinflammatory phenotypes, with increased *Tnf*α, *Il-6*, and *Il-1*β but decreased *Arg1*, *Cd206*, and *Il-10* expression (Fig. 3D). The mRNA expression of proinflammatory cytokines (e.g., *Tnf*α and *Il-6*) was also up-regulated in *Irg1*^−/−^ BMDMs compared to *Irg1*^+/+^ controls after B16-F10-TCM treatment (fig. S9B). In accord, Tnfα or Il-6 protein secretion was increased in *Irg1*^−/−^ BMDMs compared to *Irg1*^+/+^ controls after B16-F10-TCM treatment (fig. S9C). Flow cytometry analysis revealed that *Irg1*^−/−^ BMDMs exhibited higher levels of iNOS than *Irg1*^+/+^ cells after either coculture with B16-F10 tumor cells or treatment with B16-F10-TCM (fig. S9, D and E). The M2-like macrophage marker Cd206, however, did not differ significantly between *Irg1*^+/+^ and *Irg1*^−/−^ BMDMs after either coculture with B16-F10 tumor cells or treatment with B16-F10-TCM (fig. S9, D and E). On the other hand, genes enriched in Vegfa^+^ macrophages as identified by scRNA-seq (fig. S7A), were commonly down-regulated in *Irg1*^−/−^ BMDMs compared to *Irg1*^+/+^ controls after B16-F10-TCM treatment (fig. S9F). These results thus suggest that *Irg1*-deficient macrophages exhibit altered characteristics, including more proinflammatory features but reduced proangiogenic potential, which are associated with tumor suppressive functions.

Of note, *Irg1* mRNA expression in wild-type BMDMs was induced at 6 hours after coculture with B16-F10 tumor cells, and higher mRNA expression of proinflammatory genes and those involved in antigen presentation and chemotaxis was observed in *Irg1*^−/−^ BMDMs at 12 hours after B16-F10 coculture (Fig. 3E). These results thus indicate an association between IRG1 induction and IRG1/ITA-mediated suppression of genes linked to proinflammatory response and antigen presentation, reaffirming the anti-inflammatory role of IRG1.

Chemokines play a paramount role in regulating the infiltration of different immune cells into tumors (*23*), and, as such, these molecules affect tumor immunity and influence therapeutic outcomes in patients. The enriched chemokine signaling pathways in intratumoral monocytes/macrophages in *Irg1*^−/−^ mice attracted our attention (Fig. 3A). *Irg1* deficiency led to up-regulation of T cell chemotaxis genes (e.g., *Cxcl9* and *Cxcl10*) in isolated TAMs or TCM-treated BMDMs (Fig. 3, D and E). To determine whether Irg1 affects the ability of macrophages to facilitate T cell chemotaxis, we developed a transwell system in which BMDMs were cocultured with B16-F10 tumor cells or treated with B16-F10-TCM (Fig. 3F). In this coculture system, *Irg1*^−/−^ BMDMs promoted the migration of CD8^+^ T cells, which were isolated from the spleen of wild-type mice, more efficiently than *Irg1*^+/+^ macrophages. This effect was, however, abrogated by the CXCR3-specific antagonist SCH546738, which blocks the CXCL9, CXCL10, CXCL11/CXCR3 axis and thus T cell migration (*24*). Administration with SCH546738 reversed the effect of *Irg1* deficiency on facilitating T cell chemotaxis and diminished the tumoricidal effect in vivo (Fig. 3, G and H), suggesting the potential role of IRG1 in limiting T cell trafficking into the tumor.

### *Irg1*-deficient macrophages promote T cell trafficking in a Tet2-dependent manner

**Fig. 4.**
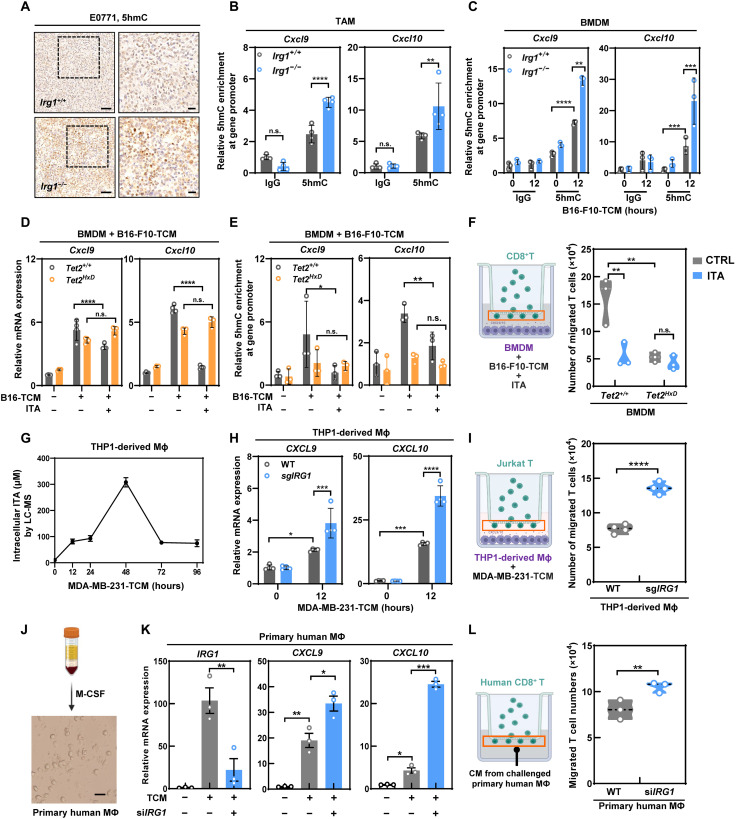
*Irg1*-deficient macrophages promote T cell trafficking in a Tet2-dependent manner. (**A**) Representative images of 5hmC staining in serial sections of E0771 breast tumors as described in Fig. 1B. Scale bars of low and high magnification represent 50 and 20 μm, respectively. (**B** and **C**) 5hmC enrichment at the promoter regions of indicated genes was determined by hMeDIP-qPCR using TAMs isolated from B16-F10 melanoma (B) or BMDMs treated with or without B16-F10-TCM (C). (**D** to **F**) Gene expression (D) and 5hmC enrichment (E) of indicated genes in BMDMs from *Tet2*^+/+^ and *Tet2*^HxD^ mice. CD8^+^ T cells that migrated into the lower chambers were counted (F) (**G** to **I**) THP1 cells were differentiated to macrophages and then were stimulated with MDA-MB-231-TCM for indicated time. Intracellular ITA was detected by liquid chromatography–mass spectrometry (LC-MS) (G). Gene expression was determined by qRT-PCR (H). Jurkat T cells migration were counted (I). (**J** to **L**) Human PBMCs were differentiated into macrophages. Scale bar, 50 μm (J). *IRG1* was depleted by siRNA and the mRNA expression of indicated genes was examined by qRT-PCR after MDA-MB-231-TCM stimulation for 12 hours (K). CM from those macrophages was placed in the lower chambers. Primary CD8^+^ T cells were isolated and then placed in the upper chambers and their migration into the lower chambers were counted (L). Data shown are replicates from three donors. The *P* values were calculated by two-way ANOVA (B), (C), (D), (E), (F), and (H); one-way ANOVA (K); and unpaired, two-tailed Student’s *t* test (J). *P < 0.05, **P < 0.01, ***P < 0.001, and ****P < 0.0001.

We previously showed that CXCL9, CXCL10, and CXCL11, three chemokines that attract T cells through binding to their receptor CXCR3 on T cells, are direct targets of TET2 (*25*). When Tet-catalyzed 5-hydroxymethylcytosine (5hmC) production was examined in engrafted tumor tissues, we found that 5hmC staining intensity was increased in tumors from *Irg1*^−/−^ than *Irg1*^+/+^ mice (Fig. 4A). Furthermore, 5hmC mapping showed that its enrichment at the promoter regions of chemokine genes (e.g., *Cxcl9*, *Cxcl10*, *Ccl2*, *Ccl3*, *Ccl4*, and *Ccl5*) was increased in TAMs from *Irg1*^−/−^ tumor-bearing mice than *Irg1*^+/+^ controls (Fig. 4B and fig. S10A). Increased 5hmC at these gene promoters was also observed in *Irg1*^−/−^ BMDMs treated with B16-F10-TCM (Fig. 4C and fig. S10B). We isolated BMDMs from catalytically inactive *Tet2*^HxD^ knock-in (KI) mutant (*9*) or wild-type mice and and then treated these macrophages with B16-F10-TCM either alone or together with ITA. The *Tet2*^HxD^ KI mutant contains H1295Y and D1297A double substitution mutations in mouse Tet2, which are equivalent to H1382 and H1384 in human TET2, respectively (*26*), and disrupt the binding with the essential cofactor Fe^2+^. We found that ITA suppressed the effect of B16-F10-TCM on up-regulating *Cxcl9/10* mRNA expression in wild-type BMDMs, but not in catalytically inactive Tet2^HxD^ macrophages (Fig. 4D). Consistently, 5hmC enrichment at the promoter of *Cxcl9/10* and selected chemokine genes was inhibited by ITA treatment in wild-type, but not in Tet2^HxD^ macrophages (Fig. 4E and fig. S10C). As a result, ITA prevented the effect of wild-type, but not Tet2^HxD^ BMDMs on promoting CD8^+^ T cell migration in the coculture transwell system (Fig. 4F).

In agreement with our findings in murine macrophages, human IRG1 protein expression was induced in THP1-derived macrophages upon stimulation with TCM from breast cancer cell line MDA-MB-231, with intracellular ITA reaching as high as 300 μM (Fig. 4G and fig. S10D). In these MDA-MB-231-TCM stimulated human macrophages, deletion of *IRG1* (fig. S10E) led to up-regulation of *CXCL9/10* and other chemotaxis genes (Fig. 4H and fig. S10F), thereby promoting the migration of Jurkat T cells in the transwell system (Fig. 4I). Furthermore, we also isolated human peripheral blood mononuclear cells (PBMCs) and differentiated them into macrophages ex vivo (Fig. 4J). As expected, *IRG1* mRNA expression was remarkably induced by MDA-MB-231-TCM in primary human macrophages (Fig. 4K). *IRG1* depletion by small interfering RNA (siRNA) significantly up-regulated *CXCL9/10* expression in primary human macrophages upon stimulation with MDA-MB-231-TCM (Fig. 4K). In accord, the conditioned medium (CM) from *IRG1-*deficient human macrophages facilitated the migration of CD8^+^ T cells in the transwell system (Fig. 4L). Together, these results in mouse and human cells suggest that IRG1/ITA affect the role of TAMs in influencing T cell trafficking through regulating TET2-CXCL9/10/11-CXCR3 axis.

### Deletion of *Irg1* in mice enhances the efficacy of anti–PD-(L)1 immunotherapy

**Fig. 5.**
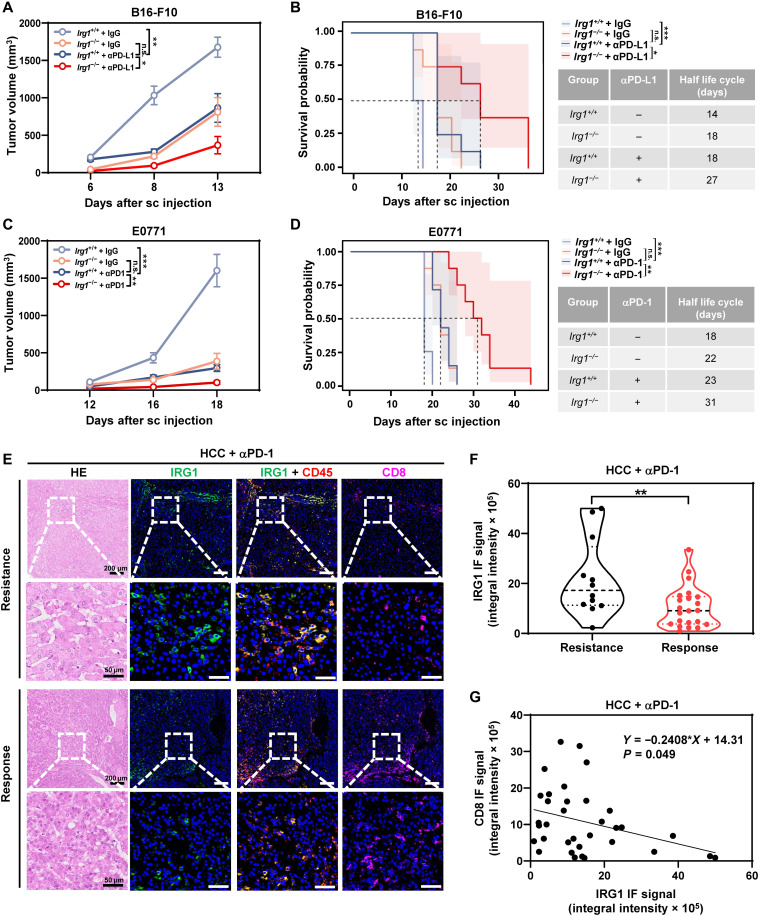
Loss of *Irg1* enhances the efficacy of anti–PD-(L)1 immunotherapy. (**A** and **C**) Tumor growth of B16-F10 or E0771 was measured in *Irg1*^+/+^ and *Irg1*^−/−^ mice treated with αPD-(L)1 antibody or IgG control (*n* = 8 mice per group). Data shown are means ± SEM, and the *P* values were calculated using two-way ANOVA. (**B** and **D**) Overall survival of mice as described in (A) and (C). The *P* values were calculated using log-rank (Mantel-Cox) test. (**E**) Human tumor samples were collected from patients with hepatocellular carcinoma (HCC) after receiving αPD-1 immunotherapy (*n* = 34). The immunostaining of IRG1, CD45, and CD8 was performed, and the signal intensity was quantified and analyzed. Representative images of indicated staining in serial sections of tumors are shown. Scale bars of low and high magnification represent 200 and 50 μm, respectively. HE, hematoxylin and eosin. (**F**) IRG1 signal in indicated groups was quantified, and the *P* values were calculated using unpaired, two-tailed Student’s *t* test. (**G**) Pearson correlation analysis of the relationship between CD8 signal intensity and IRG1 intensity in cancer tissues from patients with HCC. *P < 0.05, **P < 0.01, and ***P < 0.001. IF, immunofluorescence.

Supporting the notion that targeting *IRG1* may skew TAMs into an antitumor mode that enhances T cell–mediated tumor killing, the percentage of tumor-infiltrating CD8^+^ T cells was increased in *Irg1*^−/−^ mice. However, their cellular production of IFN-γ or tumor necrosis factor–α (TNF-α) did not differ in the TME of B16-F10 tumors between *Irg1*^−/−^ and *Irg1*^+/+^ mice (fig. S11), indicating that Irg1 may not directly affect the tumor killing function of T cells. Next, we investigated the functional consequences of *Irg1* loss on cancer responses to T cell–based immunotherapy. To this end, B16-F10 melanoma cells were inoculated subcutaneously into *Irg1*^+/+^ and *Irg1*^−/−^ mice that were then subjected to PD-L1 blockade. We found that the growth of B16-F10 tumor in *Irg1*^−/−^ mice (without PD-L1 blockade) was reduced to the extent achieved by the anti–PD-L1 in *Irg1*^+/+^ mice, extending the life span from 14 days in untreated *Irg1*^+/+^ mice to 18 days in *Irg1*^−/−^ mice or *Irg1*^+/+^ mice treated with PD-L1 antibody (Fig. 5, A and B). Notably, the combination of anti–PD-L1 treatment and *Irg1* deficiency achieved the most significant antitumor effect and longest survival, with the mean life span extended to 27 days (Fig. 5, A and B). In another syngeneic tumor model, the E0771 tumor growth in *Irg1*^−/−^ mice (without PD-1 blockade) was comparable to that in anti–PD-1–treated *Irg1*^+/+^ mice (Fig. 5, C and D). The combination of anti–PD-1 and *Irg1* deficiency showed the most effective tumor inhibition and prolonged survival, with the mean life span being extended from 22 and 23 days in *Irg1*^−/−^ mice (without PD-1 blockade) or anti–PD-1–treated *Irg1*^+/+^ mice, respectively, to 31 days (Fig. 5, C and D).

To gain more insight into human relevance, we examined and found that IRG1 expression was readily detected in patients with hepatocellular carcinoma (HCC) after immunotherapy (Fig. 5E). In a study cohort consisting of 34 patients with HCC (table S2), we found that IRG1 staining signal was higher in αPD-1–resistant HCC patients (*n* = 12) than responsive patients (*n* = 22) (Fig. 5, E and F). In agreement with our data in mouse tumor models, IRG1 expression exhibited a negative correlation with tumor-infiltrating CD8^+^ T cells in clinical samples of patients with HCC after immunotherapy (Fig. 5, E and G), further suggesting that targeting IRG1/ACOD1 represents a promising strategy for enhancing the efficacy of cancer immunotherapy.

### *Irg1* deficiency in macrophages, but not neutrophils, contributes to the enhanced antitumor immunity

**Fig. 6.**
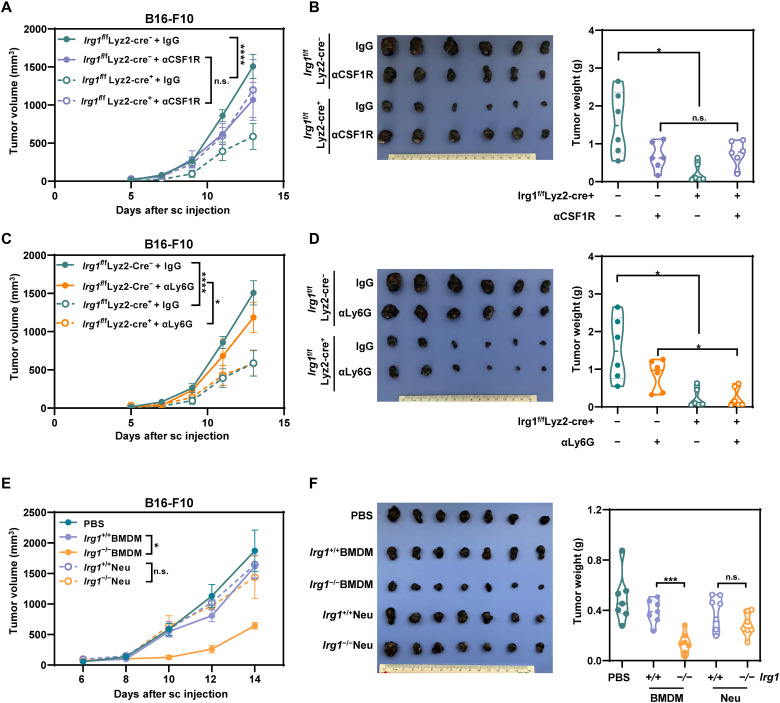
*Irg1* deficiency in macrophages contributes to the tumor phenotype. (**A**) B16-F10 tumor growth in myeloid-specific *Irg1*-KO mice and WT controls with or without αCSF1R antibody treatment was measured (*n* = 6 per group). Data shown are means ± SEM, and the *P* values were calculated using two-way ANOVA. (**B**) Tumors described in (A) were harvested at day 14 after B16-F10 inoculation, and weight was measured. Data are shown by violin plot, and the *P* values were calculated using one-way ANOVA. (**C**) B16-F10 tumor growth in myeloid-specific *Irg1*-KO mice and WT controls with or without αLy6G antibody treatment was measured (*n* = 6 per group). Data shown are means ± SEM, and the *P* values were calculated using two-way ANOVA. (**D**) Tumors described in (C) were harvested at day 14 after B16-F10 inoculation, and weight was measured. Data are shown by violin plot, and the *P* values were calculated using one-way ANOVA. (**E**) Mice were injected intratumorally with *Irg1*^+/+^ or *Irg1*^−/−^ BMDMs or neutrophils. B16-F10 tumor volume was measured at indicated time points (*n* = 7 per group). Data shown are means ± SEM. The *P* values were calculated using two-way ANOVA. (**F**) Tumors described in (E) were harvested at day 14 after B16-F10 inoculation, and weight was measured. Data are shown by violin plot, and the *P* values were calculated using one-way ANOVA. *P < 0.05; ***P < 0.001; ****P < 0.0001; n.s., not significant.

Beside TAM, our scRNA-seq results revealed that *Irg1* was also expressed in tumor-associated neutrophils (TANs) in the TME of B16-F10 tumors (fig. S6D). To decipher which *Irg1*-expressing cell population, macrophages or neutrophils, is primarily responsible for the observed tumor phenotype, we applied the antibody against CSF1R and Ly6G to deplete TAMs and TANs, respectively, in tumor-bearing mice as previously described (*27*). The injection of αCSF1R antibody led to a depletion of TAMs by ~70% in Irg1^f/f^Lyz2-cre^+^ mice (fig. S12A) and abrogated the antitumor effect of *Irg1-*KO myeloid cells (Fig. 6, A and B). The injection of αLy6G antibody led to a depletion of TANs by ~80% in Irg1^f/f^Lyz2-cre^+^ mice (fig. S12B) but had little effect on tumor phenotype (Figs. 6, C and D). Furthermore, we conducted adoptive transfer experiments by intratumoral injection of macrophages or neutrophils into B16-F10 tumor-bearing mice (fig. S12C) as previously described (*28*, *29*). The tumor-killing effect was observed in mice receiving *Irg1*^−/−^ BMDMs, but not in those animals receiving *Irg1*^−/−^ neutrophils (Fig. 6, D and E). Collectively, these results indicate that targeting *Irg1* in macrophages, but not in neutrophils, inhibits tumor growth.

**Fig. 7.**
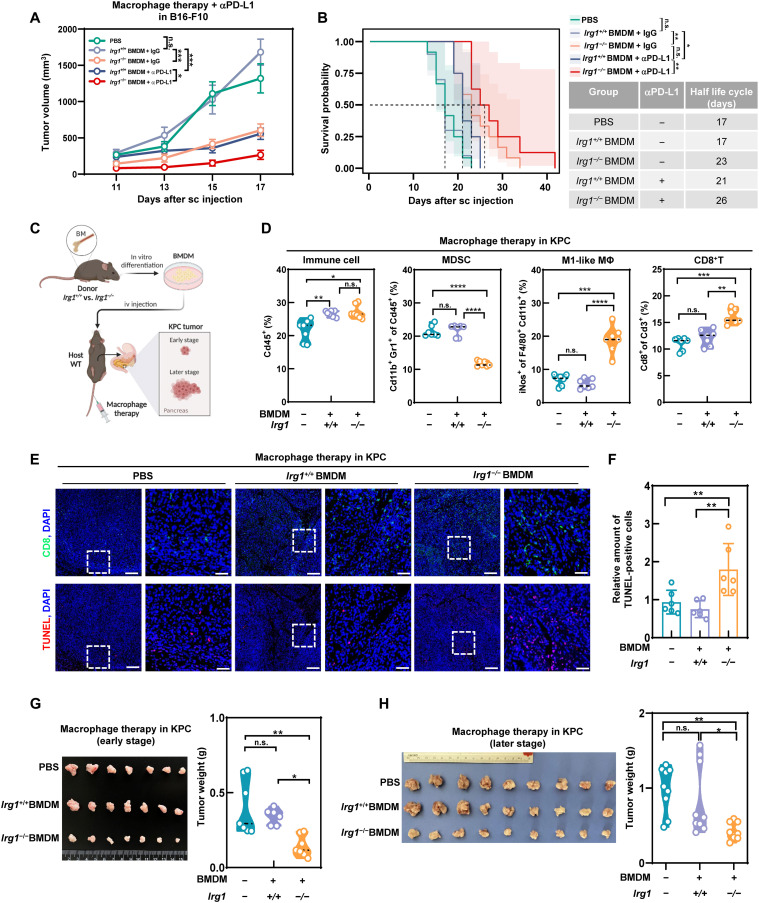
The adoptive transfer of Irg1-deficient macrophages inhibits tumor growth. (**A**) Mice were injected intratumorally with *Irg1*^+/+^ or *Irg1*^−/−^ BMDMs and intraperitoneally with αPD-L1 antibody at the same time. B16-F10 tumor volume was measured at indicated time points (*n* = 8 to 12 per group). Data shown are means ± SEM. The *P* values were calculated using two-way ANOVA. (**B**) Overall survival of mice as described in (A). The *P* values were calculated using log-rank (Mantel-Cox) test. (**C**) Experimental schematics of macrophage adoptive transfer into wild-type (WT) mice bearing KPC tumors. The recipients were intravenously injected with PBS and *Irg1*^+/+^ or *Irg1*^−/−^ BMDMs at different stages of tumor development. (**D**) The percentages of immune cells (Cd45^+^), MDSC (Cd45^+^Cd11b^+^Gr1^+^), M1-like macrophages (Cd11b^+^F4/80^+^ iNos^+^), and CD8^+^ T cells (Cd3^+^Cd8^+^) in KPC tumors at a relatively early stage, as described in (C). The *P* values were calculated by one-way ANOVA. (**E** and **F**) Representative images of IF staining of CD8^+^ and TUNEL^+^ cells in KPC tumors at a relatively early stage, as described in (C). Scale bars of low and high magnification represent 200 and 50 μm, respectively. The quantification of average cell number with SD from six random HPFs is shown. DAPI, 4′,6-diamidino-2-phenylindole. (**G** and **H**) KPC tumors were harvested and weighted after the macrophage adoptive transfer experiment at both early and later stages of tumor development, as described in (C) (*n* = 7 to 9 per group). The *P* values were calculated by one-way ANOVA. *P < 0.05, **P < 0.01, ***P < 0.001, and ****P < 0.0001.

To further enhance the tumor-killing effect of *Irg1*^−/−^ BMDMs, we combined the macrophage adoptive transfer assay together with anti–PD-L1 treatment in wild-type mice bearing B16-F10 melanoma. Notably, the tumor-killing effect of *Irg1*^−/−^ BMDM adoptive transfer was almost identical to anti–PD-L1 treatment in control mice receiving *Irg1*^+/+^ BMDMs. The adoptive transfer of *Irg1*^−/−^ BMDMs in combination with PD-L1 blockade exhibited the most effective tumor inhibition and prolonged survival in wild-type recipients (Fig. 7, A and B).

Last, we examined the effect of Irg1 deficiency in macrophages on the growth of pancreatic tumors, which are known to be resistant to T cell–based immunotherapy. Anti–PD-1 treatment failed to suppress KPC tumor growth in *Irg1*^+/+^ mice (fig. S13A). In agreement with our earlier finding (Fig. 1C), KPC tumor growth was remarkably retarded in *Irg1*^−/−^ mice compared with *Irg1*^+/+^ mice. PD-1 blockade exhibited a tendency to further decrease KPC tumor growth in *Irg1*^−/−^ mice but failed to reach statistical significance, even with more proinflammatory TAMs and increased infiltration of CD8^+^ T cells (fig. S13, A and B). Then, the macrophage adoptive transfer was conducted by intravenous injection of *Irg1*^−/−^ or *Irg1*^+/+^ BMDMs into mice bearing KPC tumors at different stages (Fig. 7C). To compare the recruitment of macrophages into engrafted KPC tumors, *Irg1*^−/−^ or *Irg1*^+/+^ BMDMs were labeled with fluorescein isothiocyanate (FITC)–conjugated beads, as previously reported (*30*). These FITC-labeled macrophages were then intravenously injected into mice implanted with KPC tumor cells (1 × 10^7^ per mouse) (fig. S14A). We found that the transferred macrophages comprised ~40% of total intratumoral macrophages in the TME of KPC tumors receiving either *Irg1*^−/−^ or *Irg1*^+/+^ macrophages (fig. S14B), suggesting that Irg1 does not affect the macrophage trafficking to and infiltration into KPC tumor sites.

Flow cytometry analysis revealed that the populations of M1-like TAMs (iNOS^+^F4/80^+^Cd11b^+^) and CD8^+^ T cells (Cd8^+^Cd3^+^) were significantly increased in KPC tumors receiving *Irg1*^−/−^ macrophages compared to *Irg1*^+/+^ controls (Fig. 7D). Immunofluorescent staining in KPC tumors in mice infused with *Irg1*^−/−^ macrophages confirmed more iNOS^+^ or CD8^+^ cells (Fig. 7E and fig. S14C) and terminal deoxynucleotidyl transferase–mediated deoxyuridine triphosphate nick end labeling (TUNEL)–positive apoptotic cells (Fig. 7, E and F). Moreover, the population of MDSCs (Gr1^+^Cd11b^+^Cd45^+^), which are known to be significantly expanded and contribute to ineffective therapies in pancreatic cancer (*31*), was decreased in KPC tumors receiving *Irg1*^−/−^ macrophages (Fig. 7D). In addition, we also checked the tumor vasculature and found a decrease in CD31 or VCAM1 staining in KPC tumors receiving *Irg1*^−/−^ macrophages compared to *Irg1*^+/+^ controls (fig. S15, A and B), suggesting that targeting *Irg1* in macrophages may suppress angiogenesis that is consistent with the scRNA analysis (Fig 2C and fig. S7) and decrease pancreatic tumor malignancy. In accord, the number of Ki67^+^ proliferating cells was decreased in KPC tumors receiving *Irg1*^−/−^ macrophages compared to *Irg1*^+/+^ controls (fig. S15C). As a result, the infusion of *Irg1*^−/−^ macrophages suppressed the growth of KPC tumors at both early (10 days after inoculation) and later stages (21 days after inoculation), resulting in a 58.8 and 50.0% decrease in tumor weight, respectively, as compared to those receiving *Irg1*^+/+^ macrophages (Fig. 7, G and H). Together, these data suggest that *Irg1*-deficient macrophages represent a potent cell therapy strategy for cancer treatment even in pancreatic tumors that are resistant to T cell–based immunotherapy.

## DISCUSSION

The present study reveals a mechanism by which tumor cells escape the immunosurveillance through inducing IRG1 expression in TAMs to create an immunosuppressive environment. This is evidenced by the expression of IRG1 in TAMs of human and mouse tumors, by cell (co)culture studies demonstrating that tumor cells or tumor-secreted factors induce IRG1 expression through NF-κB activation, by genetic studies in mice showing that deletion of *Irg1* in TAMs reduces tumor growth and enhances the efficacy of PD-1/PD-L1 blockade, and by human data showing a negative correlation of IRG1 expression with tumor-infiltrating CD8^+^ T cells and HCC patient response to immunotherapy. We believe that the IRG1-mediated escape of immunosurveillance is a broad mechanism, as deletion of *Irg1* suppresses tumor growth in multiple syngeneic mouse models, including melanoma, colon, breast, and pancreatic cancers.

We provide ex vivo and in vivo evidence that IRG1 can change TAM functionality toward proangiogenic features and limit proinflammatory features (fig. S16). This is demonstrated by reduced expression of proangiogenic genes in *Irg1*-deficient macrophages stimulated with tumor CM, as well as decreased angiogenesis in syngeneic pancreatic tumors after receiving *Irg1-*deficient macrophage therapy. On the other hand, increased expression of proinflammatory genes in TAMs from tumor grown in *Irg1*-deficient mice, such as genes involved in immunogenic antigen presentation and T cell chemotaxis, further supports the notion that IRG1 induction favors tumor cells to escape the antitumor immunity via skewing TAM polarization. At the molecular level, ITA achieves its anti-inflammatory function and suppresses proinflammatory features in macrophages, in part, through inhibition of Tet2-catalyzed DNA demethylation (*9*). Besides Tet inhibition, ITA is known to cause SDH inhibition that results in succinate accumulation and mitochondrial respiration (*32*, *33*), protein alkylation at cysteine residues that induces the electrophilic stress response mediated by NRF2 (nuclear factor erythroid 2-related factor 2) and IκBζ [inhibitor of NF-κB (nuclear factor κB) ζ] (*8*, *34*), impairment of aerobic glycolysis (*35*), and increased lysosomal biogenesis (*36*). The contribution of each of these mechanisms to the role of IRG1 in mediating tumor escape of immunosurveillance needs further clarification.

Zhao *et al.* (*37*) recently reported that intracellular ITA was higher in polymorphonuclear myeloid-derived suppressor cells (PMN-MDSCs) than that in naïve bone marrow cells, and they proposed non–cell-autonomous mechanism in which ITA produced by myeloid cells is secreted out, up-taken by T cells, and then attenuates CD8^+^ T cell proliferation and function Differently, the data presented here support a cell-autonomous mechanism in which ITA produced by myeloid cells regulates gene expression within TAMs, in part, via inhibiting Tet2, thereby changing TAM inflammatory features and their role in recruiting CD8^+^ T cells into tumor sites. In our mouse tumor models, we cannot detect the intracellular accumulation of ITA in tumor-infiltrating lymphocytes (CD45^+^CD11b^−^) (fig. S4B). Moreover, we provide in vivo evidence that *Irg1* deficiency may not directly affect the tumor killing function of T cells (fig. S11), whereas the study by Zhao *et al. *(*37*) relied on mostly in vitro study using exogenously added supraphysiological high concentration of ITA. It therefore remains to be determined whether ITA is sufficiently uptake by the T cells and regulate T cell function in vivo. Despite different cell types and mechanisms of ITA action, non–cell-autonomous T cell proliferation versus cell-autonomous macrophage polarization, the study by Zhao *et al. *(*37*) and our current study show that deletion of *Irg1* in mice enhances antitumor immunity.

There are two clinical implications of this study. First, it identifies ACOD1 as a potential target for developing immunotherapy drugs. The current antibody-based immune checkpoint blockades, in addition to their limited efficacy in most cancer types, are less convenient to administer because of its intravenous injection and take a long time to resolve adverse effect due to the long half-life of antibody. We show that *Irg1-*deficient mice, which develop normally, are resistant to several types of synergetic tumors. The antitumor effect conferred by *Irg1* loss is similar to that from administration of anti–PD-(L)1 immunotherapy. The combination of *Irg1* deficiency and PD-1/PD-L1 blockade can collaborate to enhance the antitumor immunity, suggesting that targeting ACOD1 can be effective and convenient for monotherapy as well as adjuvant therapy with current PD-(L)1 blockades for treating different cancer types. Mechanistically, the coordination between an ACOD1 inhibitor and PD-(L)1 blockades can be explained by the orthogonality of their respect target cells and mechanisms of action. While ACOD1 inhibitors target monocytes/macrophages to reprogram TAM polarization and promote antigen presentation and T cell trafficking, PD-(L)1 blockades target and reenergize CD8^+^ T cells and restore their antitumor effector functionality.

Another clinical implication of our study is that *IRG1*-deficient macrophages could potentially be used for cell therapy for cancer. Although the adoptive transfer of T cells and CAR-T therapy have produced clinical efficacy in the treatment of blood malignancies (*38*), their applications have so far failed to demonstrate a similar efficacy in solid tumors (*39*). Much of the failures can be attributed to the lack of T cell trafficking into tumor sites and the immunosuppressive milieu suppressing the cytotoxic activity of T cells. Increasing evidence suggests that the adoptive therapy of genetically engineered monocytes and macrophages can overcome some of these limitations, as they penetrate tumors more efficiently (*40*) and, in their proinflammatory state, exhibit tumoricidal activity in solid tumors (*41*). In this study, we show that the infusion of *Irg1*-deficient macrophages, but not neutrophils, into tumor-bearing mice exerts potent tumor-killing effects and also enhances the efficacy of anti–PD-(L)1 immunotherapy in the melanoma model (Fig. 6, E and F, and Fig. 7, A and B), in which most of TAMs should be from recruited monocytes. Differently, both recruited monocytes and tissue-resident macrophages are major sources of TAMs in KPC pancreatic tumors (*42*). It is known that monocyte-derived TAMs (recruited TAMs) play potent roles in antigen presentation, while resident TAMs are responsible for remodeling extracellular matrix. We think that *Irg1* deletion should predominantly occur in “recruited” TAMs but not “resident” TAMs in the orthotopic KPC model (Fig. 7C). Whether transferred TAMs would affect resident TAMs and thereby change the extracellular matrix to affect the recruitment of MDSCs, as seen in the KPC pancreatic tumor model but not in the melanoma model, still needs further investigation. Nevertheless, our current findings, together with the recent development in CAR-macrophage engineering (*40*) and induced pluripotent stem cell–derived macrophages (*43*), suggest that adoptive transfer of *IRG1*-deficient macrophages merits exploration as a cell therapy strategy for solid tumors.

## MATERIALS AND METHODS

### Analysis of publicly available datasets

The genome-wide transcriptome data quantified as fragments per kilobase per million were downloaded from TCGA (*44*). We used the data from 18 cancer types, all of which had profiled enough numbers of samples for both cancer and normal tissues. Data include BLCA, BRCA, cholangiocarcinoma (CHOL), COAD, ESCA, HNSC, KICH, kidney clear cell carcinoma (KIRC), KIRP, liver hepatocellular carcinoma (LIHC), lung adenocarcinoma (LUAD), LUSC, PAAD, prostate adenocarcinoma (PRAD), rectum adenocarcinoma (READ), STAD, thyroid carcinoma (THCA), and UCEC. For the SKCM, the cancer samples were collected from TCGA, whereas the normal control samples were collected from Genotype-Tissue Expression (GTEx) (*45*). The mRNA expression of *IRG1* gene was compared between normal and cancer samples. Wilcoxon rank sum test was used to determine the significance of difference for these 19 cancer types. EPIC (*46*) was used to calculate the immune cell fractions for all the samples. The cell fraction of TAMs was identified, and Spearman rank correlation test was used to determine the significance of correlation between immune cell fractions and *IRG1* expression level.

### Clinical sample collection

Samples from patients with HCC were collected by the Department of Liver Surgery, Zhongshan Hospital of Fudan University, and by the Department of General Surgery, Huashan Hospital of Fudan University. Informed consent was received from each patient before surgery. The procedures related to human subjects were approved by the Ethics Committee of Zhongshan Hospital (project approval number Y2020-622) and Huashan Hospital (project approval number 2020 M-010). Information about clinical samples used in this study is listed in table S2.

### Animals and study approval

*Irg1*^−/−^ mice (JAX stock no. 029340) were purchased from the Jackson Laboratory. Animals were backcrossed for more than seven generations onto the C57BL/6J background and were maintained at Shanghai Research Center for Model Organisms. Myeloid cell–specific *Irg1*-deficient mice (*Irg1*^f/f^, *Lyz-Cre*) on C57BL/6J background was generated by Shanghai Model Organisms Center Inc. and were maintained at the Center for New Drug Evaluation and Research, China Pharmaceutical University (Nanjing, China). Tet2^HxD^ KI–mutant mice on C57BL/6J background were constructed by using CRISPR-Cas9 system, which were provided by J. Chen and S. Gao (School of Life Sciences and Technology, Tongji University, Shanghai) and were maintained at GemPharmatech Co. Ltd. Analysis was performed on 6- to 10-week-old female mice obtained from the abovementioned breeding. Animals were given unrestricted access to a standard diet and tap water. Animal experiments were performed at Fudan Animal Center in accordance with animal welfare guidelines, and the procedures were approved by the Ethics Committee of the Institutes of Biomedical Sciences (IBS), Fudan University.

### Mouse genotyping

For the genotyping of *Irg1*^−/−^ mice, we followed the instruction from the Jackson Laboratory. Briefly, three primers were used for PCR followed by agarose gel electrophoresis: GTG GGG AGG GGA ACT ATG AG (forward primer for both wild-type and mutant Irg1), ATT TGG AGG AAC CCC ATG AC (reverse primer for wild-type), and CAG CCT CTA AGC CAG ACA GC (reverse primer for mutant). The sizes of PCR fragments are 157 and 220 base pairs (bp) for the wild-type and mutant Irg1, respectively.

For the genotyping of myeloid cell–specific *Irg1*-deficient mice (Irg1^f/f^, Lyz2-Cre), two primers were used for the genotyping of flox: CAGGAAGGCCAGTGCTCA GTAATC (forward primer) and ACCTCCTCGCACCCCCTTTGTATG (reverse primer). The sizes of PCR fragments are 331 and 388 bp for the wild-type and mutant Irg1, respectively. Moreover, four primers were used to genotype Lyz2-Cre: GGAGGATGC TTAAATAGCAGG (transgene forward primer), CATCACTCGTTGCATCGACC (transgene reverse primer), AGTGGCCTCTTCCAGAAATG (internal positive control forward primer), and TGCGACTGTGTCTGATTTCC (internal positive control reverse primer). The sizes of PCR fragments are 150 and 521 bp for Cre^+^ and control, respectively.

For the genotyping of *Tet2*^HxD^ mice, two primers were used: CTGGATATCTGC AGAATTCACCTTACTACACCGAGAAGCCT and GGTACCGAGCTCGGATCCACCCTTCCTAG GTG ACAGGTGAC. The PCR products were cloned to pCDNA3.1 digested by EcoR I and BamH I. The constructs were sequenced and compared with wild-type *Tet2* DNA sequence to confirm the two mutant sites as previously reported (*9*).

### Cell culture

HEK293T cells [American Type Culture Collection (ATCC), CRL-3216], B16-F10 cells (ATCC, CRL-6475), MC38 cells (Kerafast, catalog no. ENH204), and E0771 cells (ATCC, CRL-3461) were commercially purchased. FC1242 KPC cells were gifted from L. Fei (Fudan University). Tumor cells were maintained in Dulbecco's modified Eagle's medium (DMEM) containing 10% fetal bovine serum (FBS) (ExCell Bio, FSD500), 1% penicillin/streptomycin antibiotics.

For differentiation of BMDMs, mice were euthanized in a 5% CO_2_ chamber and death was confirmed by cervical dislocation. Bone marrow was harvested from the femur and the tibia and differentiated in DMEM [containing 10% fetal calf serum (FCS), 1% penicillin/streptomycin, and macrophage colony-stimulating factor (M-CSF) (100 ng/ml)] for 7 days. Then, 1 × 10^6^ BMDMs/ml were cocultured with tumor cells or treated with tumor-derived CM for different time points.

### Tumor cell–conditioned medium 

To prepare for TCM, B16-F10, E0771, MC38, or FC1242 tumor cells were seeded with 10 ml of medium in 10-cm dish, and the culture medium were collected after 24 hours followed by filtration using 0.45-μm filter (Corning) and were then stored at −20°C.

### Generation of syngeneic mouse tumor models

For generation of the syngeneic mouse models of melanoma, colon, and breast tumor, 2 × 10^5^ or 1 × 10^5^ B16-F10 cells, 1 × 10^5^ MC38 cells, and 1 × 10^5^ E0771 cells were kept in 25 μl of DMEM, respectively. The same volume of Matrigel (BD Biosciences, 356234) was added and mixed. The cells were subcutaneously injected into *Irg1*^+/+^ or *Irg1*^−/−^ mice (female, 8 to 10 weeks). Tumors were measured every 2 or 3 days, and the volume was calculated using the formula *V* = as (width by width by length)/2 as previously described (*22*). The survival rate was determined as previously reported (*47*). Briefly, mice were removed from the study if they were found dead in their cage, declining health necessitated killing, or tumor diameter exceeded 15 mm. These parameters were used to construct a Kaplan-Meier survival curve.

For the syngeneic mouse models of pancreatic tumor, 1 × 10^5^ FC1242 cells were kept in 25 μl of DMEM and were mixed with 25 μl of Matrigel (BD Biosciences). The cell suspension was then injected orthotopically into the pancreas of *Irg1*^+/+^ or *Irg1*^−/−^ mice (female, 8 to 10 weeks). At day 15 after FC1242 tumor inoculation, the pancreatic tumors were dissected, weighted, and subjected to further analysis.

For cancer immunotherapy, anti–PD-L1 (200 μg per mouse; BioXcell, BP0101) was intraperitoneally injected at days 6, 8, 10, 13, 15, and 17 after B16-F10 tumor inoculation; anti–PD-1 (200 μg per mouse; BioXcell, BE0146; or Biointron, B176401) was intraperitoneally delivered at days 10, 12, and 14 after E0771 tumor inoculation or intraperitoneally injected at days 7, 10, 13, and 16 after FC1242 KPC tumor inoculation. At indicated days after anti–PD-(L)1 treatment, tumor size was determined.

### Blockage of CD8^+^ T cell chemotaxis

Mice were intratumorally injected with the CXCR3 antagonist SCH546738 (600 mg per mouse; MedChem Express, HY-10017), starting at day 6 after tumor inoculation and then maintained by daily injection for 1 week.

### PBMC isolation and differentiation

Human peripheral blood was collected from healthy women donors (20 to 40 years old) following the protocol approved by the Ethics Committee of IBS, Fudan University (project approval number 2021-23). PBMCs were isolated from human peripheral blood following the manufacturer’s instructions. Briefly, whole blood (10 ml) was layered on 15 ml of Ficoll (Cytiva, 17144002) and spun for 22 min at 2300 rpm with the brake off to prevent disrupting the density gradient during deceleration. PBMCs were then isolated from the middle layer (*34*).

PBMCs were counted, and the cell density was adjusted to 2.5 × 10^6^ cells/ml and was then maintained in DMEM supplemented with 10% (v/v) FCS, 2 mM l-glutamine, and 1% penicillin/streptomycin. Cells were seeded into six-well plates in culture medium containing M-CSF (100 ng/ml; Novoprotein, C417) and maintained at 37°C, 5% CO_2_, for 9 days, to allow the differentiation into macrophages (*48*). The proper differentiation into macrophages was verified by the staining of CD14 and CD163. In these human macrophages, siRNA against *IRG1* (sequence: CACAGGUGAGAGAGCUGCUCAGUAA) was transfected using RNAiMAX reagent (Life Technologies, 13778150) for 12 hours, followed by another 12 hours of treatment with MDA-MB-231-TCM. The culture medium and challenged cells were collected for further analysis.

### Macrophage and neutrophil adoptive transfer

For the macrophage adoptive transfer experiment, tumor volume of 1 × 10^4^ BMDMs/mm^3^ was injected intratumorally when the tumor reached ~200 mm^3^ after B16-F10 inoculation with or without simultaneous intraperitoneal injection of anti–PD-L1 (200 μg per mouse).

Neutrophils were isolated from mouse bone marrow by percoll gradient, and the cell purity was confirmed by flow cytometry. For the neutrophil adoptive transfer experiment, 1.5 × 10^6^ neutrophils were intratumorally injected into B16-F10 tumor bearing mice at days 8 and 11 after inoculation. Later, the tumors were harvested and weighted at day 14. The same experimental setting was applied for the macrophage adoptive transfer experiment in Fig. 6 (E and F).

For macrophage adoptive transfer in KPC model, BMDMs at 1 × 10^7^/50 μl were intravenously injected at days 10 and 13 after FC1242 KPC tumor inoculation. Mice were euthanized at day 15 after tumor implantation, and the pancreatic tumors were dissected, weighted, and subjected to further analysis. At a later stage in KPC model, BMDMs at 1 × 10^7^/50 μl were intravenously injected at days 21 and 23 after KPC tumor inoculation. Mice were euthanized at day 26 after tumor implantation, and the samples were collected as described above.

### Intracellular metabolite quantification by liquid chromatography–tandem mass spectrometry

Cell number and cell diameter were measured using an automated cell counter (Countstar). C_13_-labeled ITA was added as an internal standard. Cells were fixed by immediate addition of 1 ml of 80% (v/v) chilled (−80°C) methanol. Cell extracts were analyzed by ultrahigh performance liquid chromatograph (Acquity UPLC I-Class, Waters) coupled to a Triple Quadrupole Mass Spectrometer (Xevo TQ-XS, Waters). Cells were assumed to have a spherical shape, and intracellular metabolite concentrations were calculated using external standard curves and taking into account cellular diameter (*d*, micrometers) and cell number using the following equation: (metabolite) = metabolite quantity (moles)/[(4/3000)π(*d*/2)^3^*cell number].

### Flow cytometry

Tumors were harvested at indicated time. Briefly, 100 mm^3^ of each tumor was chopped, digested, and filtered through a 70-μm cell strainer to generate a single-cell suspension. After red blood cell lysis (Beyotime, C3702), cells were counted and plated in phosphate-buffered saline (PBS). Cell surface molecule staining was performed at 4°C for 15 to 30 min in PBS in the dark.

For intracellular staining, cells were fixed/permeabilized in 50 μl of a saponin-containing buffer (BD Biosciences, 554722) and incubated at 4°C for 30 min in the dark. Cells were then washed two times with saponin-containing buffer (BD Biosciences, 554722) and resuspended in staining buffer followed by antibody staining. Stained samples were acquired on a LSRFortessa flow cytometer (BD Biosciences). Collected data were analyzed using FlowJo software (Tree Star Inc.). The antibodies used in this study are Fixable Viability Dye eFluor 506 (Invitrogen, 65-0866-14); phycoerythrin (PE) anti-Cd45 (BD Biosciences, 553081), FITC anti-CD45 (BD Biosciences, 553079), FITC anti-Cd11b (BD Biosciences, 561688), allophycocyanin (APC) anti-Cd11b (BD Biosciences, 553312), PE anti-F4/80 (BD Biosciences, 565410), APC anti-F4/80 antibody (Invitrogen, 17–4801-82); APC anti-mouse Ly-6G (BD Biosciences, 553129), APC-Cy7 anti–Ly-6C (BD Biosciences, 560596), PE-Cy7 anti-Cd3e (BD Biosciences, 552774), APC anti-Cd3e (BD Biosciences, 561826), FITC anti-Cd8a (BD Biosciences, 553030), APC anti-Cd8a (BD Biosciences, 553035), Peridinin-Chlorophyll-Protein (PerCP)/cyanine5.5 (Cy5.5) anti-Cd80 (BioLegend, 104722), PE-Cy7 anti-Cd86 (BD Biosciences, 560582), PE anti-Cd4 (BD Biosciences, 557308), PE anti–Nk-1.1 (BD Biosciences, 557391), PE-Cy7 anti-NOS2 (type 2 nitric oxide synthase) (eBioscience, 25-5920-80), APC anti-CD206 (BioLegend, E-AB-F1135E), APC anti–Ly-6G(GR1) (MACS, 130-098-047), APC anti-Cd40 (BD Biosciences, 558695), BV421 anti–MHC-I (BD Biosciences, 749711), APC anti–MHC-II (BioLegend, 107613), PE anti–Ifn-γ (BD Biosciences, 554412), PE anti–TNF-α (BioLegend, 506305), PE-Cy7 anti–PD-1 (BioLegend, 135215), FITC anti- CD14(BioLegend, 325604), and APC anti-CD163 antibody (BioLegend, 333610).

### Cellular immunofluorescence

For visualizing mouse immune cells in the TME, tumor samples were collected and then fixed in 4% paraformaldehyde. Paraffin-embedded sections (4 μm) were deparaffinized in xylene, then rehydrated through graded alcohol, and washed briefly in tap water. To retrieve antigenicity, sections were boiled in 10 mM citrate buffer (pH 5.8) for 30 min in microwave (800 W). Sections were left to room temperature and washed briefly in PBS. Following that, sections were incubated with goat serum diluted in PBS (pH 7.4) for 1 hour at room temperature and were washed briefly in PBS. Subsequently, sections were incubated at 4°C overnight with the primary antibodies specific for CD8 [1:200 dilution; Cell Signaling Technology (CST), 98941S] or iNOS (1:200 dilution; CST, 13120), rinsed with fresh PBS, and incubated with the secondary antibody Alexa Fluor 647 goat anti-rabbit immunoglobulin G (IgG) (1:200 dilution; Thermo Fisher Scientific, A-21245) at room temperature for 1 hour in darkness, and then washed briefly in PBS. Last, sections were incubated by ProLongGold antifade reagent with 4′,6-diamidino-2-phenylindole (Invitrogen, p36931) and mounted. Immunofluorescence pictures were taken by a Leica SP8 laser scanning confocal microscope.

To examine IRG1 expression and immune cells in samples from patients with HCC after immunotherapy, the following antibodies were used: IRG1 (D6H2Y) rabbit mAb (1:200 dilution; CST, 77510s); CD8 (1:1000 dilution; Abcam, ab4055); CD45 (1:500 dilution; Abcam, ab30470); donkey anti-rabbit IgG (H+L) secondary antibody, Alexa Fluor 488 conjugate (1:200 dilution; Thermo Fisher Scientific, A-21206); and donkey anti-mouse IgG (H+L), Alexa Fluor 594 (1:200 dilution; Thermo Fisher Scientific, A-21203). Immunofluorescence pictures of stained slides were taken by OLYMPUS SLIDEVIEW VS200 and analyzed by ImageJ.

### Quantitative real-time PCR

Total RNA was extracted from cells using TRIzol (Invitrogen) as recommended by the manufacturer’s protocol. RNA was reversely transcribed using oligo(dT) primers. Diluted complementary DNA (cDNA) was then used in qRT-PCR reactions containing SYBR Premix ExTaq (TaKaRa) and gene-specific primers. The reactions were performed in the QuantStudioTM 6 Flex Real-Time PCR System (Applied Biosystems). 18*S* RNA was used as a housekeeping control. Primers used in this study are listed in table S3.

### Single-cell RNA sequencing

*Irg1*^−/−^ and *Irg1*^+/+^ mice were injected subcutaneously with 2 × 10^5^ B16-F10 cells (in 50 μl of PBS). Ten days after inoculation, tumors were harvested, and 100 mm^3^ of each tumor was collected. Tumor tissues from three mice of the same group were randomly combined into one mixed sample, and then, tissues were digested and went through 70-μm filters (BD Biosciences, 352350) to achieve single-cell suspensions. After treatment with red blood cell lysis buffer (Beyotime Biotechnology, C3702) for 5 min at room temperature, all samples were washed and resuspended in flow cytometry buffer (PBS/0.5% albumin/2 Mm EDTA). In the end, Cd45^+^ immune cells (*n* = 6000 per sample) were sorted by flow cytometry and subjected to scRNA-seq (NovelBio Bio-Pharm Technology Co.).

BD Rhapsody system was used to capture the transcriptomic information of the single cells (*49*). Single-cell capture was achieved by random distribution of a single-cell suspension across >200,000 microwells through a limited dilution approach. Beads with oligonucleotide barcodes were added to saturation so that a bead was paired with a cell in a microwell. The cells were lysed in the microwell to hybridize mRNA molecules to barcoded capture oligos on the beads. Beads were collected into a single tube for reverse transcription and ExoI digestion (New England Biolabs, M0293S). Upon synthesis, each cDNA molecule was tagged on the 5′ end (that is, the 3′ end of mRNA transcript) with a unique molecular identifier (UMI) and cell barcode indicating its cell of origin. Whole transcriptome libraries were prepared using the BD Rhapsody single-cell whole-transcriptome amplification (WTA) workflow including random priming and extension (RPE), RPE amplification PCR, and WTA index PCR. The libraries were quantified using a High Sensitivity DNA chip (Agilent) on a Bioanalyzer 2200 and the Qubit High Sensitivity DNA assay (Thermo Fisher Scientific). Sequencing was performed by sequencer (Illumina, San Diego, CA) on a 150-bp paired-end run (*49*).

Next, scRNA-seq data analysis was performed by using the NovelBrain Cloud Analysis Platform. We applied fastp with default parameter filtering the adaptor sequence and removed the low-quality reads to achieve the clean data. UMI-tools were applied to identify the cell barcode whitelist. The UMI-based clean data were mapped to mouse genome (Ensemble version 100) using STAR mapping with customized parameter from UMI-tools standard pipeline to obtain the UMI counts of each sample. Cells contained over 200 expressed genes and mitochondria UMI rate below 10% passed the cell quality filtering, and mitochondria genes were removed in the expression table. Seurat package (version 3.1.4, https://satijalab.org/seurat/) was used for cell normalization and regression, based on the expression table according to the UMI counts of each sample and percent of mitochondria rate to obtain the scaled data. Principal components analysis (PCA) was constructed on the basis of the scaled data with top 2000 high variable genes, and top 10 principals were used for t-distributed Stochastic Neighbor Embedding (t-SNE) construction and UMAP construction.

By using graph-based cluster method (resolution, 0.8), we acquired the unsupervised cell cluster result based on the PCA top 10 principal and calculated the marker genes by FindAllMarkers function with Wilcoxon rank sum test algorithm under the following criteria: (i) Ln (Fold Change) (LnFC) > 0.25, (ii) *P* < 0.05, and (iii) minimum percentage (Min.pct) > 0.1. To identify the cell type detailed, the clusters of same cell type were selected for re-t-SNE analysis, graph-based clustering, and marker analysis (*50*). To identify differentially expressed genes among samples, the function FindMarkers with Wilcoxon rank sum test algorithm was used under following criteria: (i) LnFC > 0.25, (ii) *P* < 0.05, and (iii) Min.pct > 0.1 (*50*).

### ChIP with qPCR

Chromatin immunoprecipitation (ChIP) assay was performed as described previously (*51*). Briefly, cells were cross-linked with 1% paraformaldehyde and sonicated at 4°C for 20 min (Bioruptor, low mode). Chromatin was immunoprecipitated at 4°C for 3 hours with indicated antibodies. Antibody-chromatin complexes were pulled down using protein A-Sepharose (Millipore, 16-125), washed, and eluted. After cross-link reversal and proteinase K treatment (TaKaRa, 9034), immunoprecipitated DNA was extracted using the PCR Purification Kit (QIAGEN, 28006). The following primer sequences were used: Acod1 (forward): AATGGGCTGTCTGTGAGA; Acod1 (reverse): CCAGGTCTGTCCCTTTCAC.

### Enzyme-linked immunosorbent assay

Supernatants of 5 × 10^5^ BMDM cells in six-well plates were collected at 12 hours after stimulation of B16-F10-TCM. Cytokines were assessed by enzyme-linked immunosorbent assay by using the kits of Tnf-α (DY410-05) and IL-6 (DY406-05), purchased from R&D Systems (Minuteneapolis, MN, USA), according to the manufacturer’s instructions.

### TAMs and TANs depletion assay

Myeloid-specific *Irg1*-KO mice and wild-type controls were inoculated with 1 × 10^5^ B16-F10. Then, the antibody of anti-CSF1R (500 μg per mouse, BioXcell, BE0213) or anti-Ly6G (400 μg per mouse, BioXcell, BE0075) was intraperitoneally injected from day 3 after inoculation for every 2 days. The tumors were harvested to define the depletion efficiency at day 14 by flow cytometry analysis.

### T cell migration assay

T cell migration assay was performed by using 24-well 6.5-mm transwell with 5.0 μm Pore Polycarbonate Membrane Insert. In brief, BMDMs (5 × 10^5^) were cocultured with B16-F10 tumor cells (5 × 10^5^) or challenged with B16-F10-TCM for 12 hours at the bottom chamber. Meanwhile, CD8^+^ T cells (1 × 10^6^) were isolated from the spleen of wild-type mice and suspended in 200 μl of 1% FBS–RPMI 1640 and were then placed at the top chamber with or without 100 nM SCH546738 (MCE, HY-10017). The migration of CD8^+^ T cells from the top to the bottom chamber was evaluated after incubation for 4 hours at 37°C in a 5% CO_2_ atmosphere. Cells in the bottom chambers were collected and the number of CD8^+^ T cells was counted.

In addition, PMA-differentiated THP1-derived macrophages (1 × 10^6^) were challenged with MDA-MB-231-TCM for 12 hours at the bottom chamber. Meanwhile, Jurkat T cells (1 × 10^6^) were suspended in 200 μl of 1% FBS–RPMI 1640 and were placed at the top chamber. The migration of Jurkat T cells from the top to the bottom chamber was evaluated after 4-hour incubation at 37°C in a 5% CO_2_ atmosphere.

Furthermore, human peripheral blood was subjected to EasySep Human CD8^+^ T Cell Enrichment Kit (STEMCELL Technologies, 17953) to isolate primary CD8^+^ T cells, and these T cells were placed at the top chamber. Meanwhile, the CM from primary human macrophages after stimulation with MDA-MB-231-TCM was placed at the bottom chamber. The migration of CD8^+^ T cells from the top to the bottom chamber was evaluated after 6-hour incubation at 37°C in a 5% CO_2_ atmosphere, as previously reported (*52*).

### Statistical analysis and reproducibility

Statistical analyses were performed using GraphPad Prism software version 8.0. Summarized views on data that underlie the statistical tests as well as the exact *P* values are available in the Source Data. The statistical details for each experiment are also provided in the figure legends. Generally, the statistical analyses were performed using a two-tailed Student’s *t* test for paired comparisons or one/two-way analysis of variance (ANOVA) for multiple comparisons. The data shown (unless indicated otherwise) represent the results obtained from at least triplicate independent experiments with the means ± SD or SEM. Where indicated, *P < 0.05, **P < 0.01, ***P < 0.001, and ****P < 0.0001, and n.s. denotes nonsignificant.

In this study, in vivo experiments were repeated at least two or three times, and the sample size was defined empirically or referred to previous literatures. In vitro experiments were performed including at least three biological replicates, with two to three technical replicates for each biological replicate, to confirm the reproducibility.

## Notes

View/request a protocol for this paper from *Bio-protocol*.
