# Supplementary material for: Targeting IRG1 reverses the immunosuppressive function of tumor-associated macrophages and enhances cancer immunotherapy
Source: Sci Adv. 2023 Apr 28;9(17):eadg0654. doi: 10.1126/sciadv.adg0654 (PMC10146892; doi:10.1126/sciadv.adg0654)
Supplement: Supplementary file 1 — Figs. S1 to S16 Tables S1 to S3 [file sciadv.adg0654_sm.pdf]

Science Advances
AAAS

Supplementary Materials for

Targeting IRG1 reverses the immunosuppressive function of tumor-associated macrophages and enhances cancer immunotherapy

Yu-Jia Chen *et al.*

Corresponding author: Yong Yang, yy@cpu.edu.cn; Dan Ye, yedan@fudan.edu.cn;
Lei-Lei Chen, chenleilei@fudan.edu.cn

Sci. Adv. **9**, eadg0654 (2023)
DOI: 10.1126/sciadv.adg0654

This PDF file includes:

Figs. S1 to S16
Tables S1 to S3

Fig. S1. Positive correlation between *IRG1* mRNA expression and TAM fractions in multiple types of human tumors

EPIC (Estimating the Proportions of Immune and Cancer cells) was used to calculated TAM fraction in the indicated types of tumors from the TCGA dataset. Spearman rank correlation test was used to determine the significance of correlation between TAM fraction and *IRG1* expression.

Fig. S2. *IRG1* is expressed in monocytes and macrophages in the TME of human cancer

(**A**) According to published scRNA-seq data (GEO: GSE139829) in human Uveal melanoma, the transcripts were re-analyzed and the UMAP plot of annotated cell types is shown (upper panel). Expression of *IRG1* in indicated cell types is shown by violin plot (lower panel).

(**B**) According to published scRNA-seq data (GEO: GSE154763) in 12 types of human cancer as indicated, the transcripts were re-analyzed. Expression of *IRG1* in annotated monocytes and macrophages is illustrated in the UMAP plots.

Fig. S3. Tumor cells induce *Irg1* mRNA expression in macrophages via NF-κB activation

(**A**) *Irg1* induction and ITA accumulation in BMDMs co-cultured with tumor cells. Wild-type BMDMs were co-cultured with different types of syngeneic tumor cells for 6 or 12 hours. Data shown are mean ± s.d. of four independent experiments. The p

values were calculated by unpaired, two-tailed Student's t test.

(**B**) *Irg1* induction and ITA accumulation in BMDMs stimulated with TCM. Wild-type BMDMs were treated with conditioned medium from different types of syngeneic tumor cells for 6 or 12 hours. The mRNA expression of *Irg1* and intracellular levels of ITA were determined by qRT-PCR and LC-MS, respectively. Data shown are mean ± s.d. of four independent experiments. The p values were calculated by unpaired, two-tailed Student's t test.

(**C**) B16-F10-TCM with indicated inhibitors were used to stimulate BMDMs for 6 hours. The mRNA expression of *Il-1β*, *Il-6* and *Irg1* were determined by qRT-PCR. Data shown are mean ± s.d. of four independent experiments. The p values were calculated by one-way ANOVA.

(**D**) RelA occupancy at the promoter region of *Irg1* was determined by ChIP-qPCR. Data are the mean ± s.d. of four independent experiments. The p values were calculated by two-way ANOVA.*p < 0.05; **< 0.01; *** p < 0.001; ****p < 0.0001.

Fig. S4. *Irg1* deficiency inhibits tumor growth in immune competent mice

(**A**) B16-F10 (left), MC38 (middle) and E0771(right) were s.c. injected in *Irg1*$^{+/+}$ and *Irg1*$^{-/-}$ mice (n=7-8 per group), and tumor growth was determined by the measurement of tumor volume (mm^3). Data are mean ± s.e.m., and the p values were calculated by two-way ANOVA.

(**B and C**) Indicated cell populations were purified from B16-F10 tumors in *Irg1*$^{+/+}$ or *Irg1*$^{-/-}$ mice by flow cytometry. The intracellular levels of ITA were determined by LC-

MS. Data are the mean ± s.d.

Fig. S5. Myeloid-specific Irg1 deficiency inhibits tumor growth in immune competent mice

(**A**) The verification of myeloid specific *Irg1*-KO in mice by genotyping.

(**B** and **C**) BMDMs were differentiated from *Irg1*$^{f/f}$Lyz2-cre^{+} mice and control animals, and then stimulated with LPS for 6 hours. The mRNA (B) and protein expression (C) of Irg1 was determined by qRT-PCR and western blot, respectively.

(**D** and **E**) B16-F10 (D) and KPC (E) were s.c. injected in *Irg1*$^{f/f}$ and *Irg1*$^{f/f}$Lyz2-cre^{+} mice (n=7-12 per group). Representative images of tumors are shown.

Fig. S6. scRNA-seq unveils major immune populations in the TME of B16-F10 tumor

(**A**) Sequenced cells were divided into 19 clusters, and representative genes in each cluster is shown.

(**B**) 19 clusters were assigned to 8 types of cells, whose marker genes are shown in the table.

(**C**) Dot-plot was used to show the identity of 8 types of cells.

(**D**) Expression of *Irg1* and neutrophil marker genes are shown in the UMAP plots.

Fig. S7. Developmental trajectory of monocytes and macrophage subsets in the TME of B16-F10 tumor

(**A**) Bubble heatmap was used to show 6 monocytes/macrophages subsets with the

expression of selected genes.

(**B**) KEGG analysis of curated set of transcriptomic signatures in 6 monocyte/macrophage subsets.

(**C**) Pseudotime trajectory analysis of 6 monocytes/macrophages subsets (left panel). Model of the developmental trajectory of monocyte/macrophage subsets in B16-F10 tumor (right panel).

Fig. S8. The gating strategy for flow cytometry data

Flow cytometry gating strategy for indicated myeloid and lymphoid cells in B16-F10 tumors. Briefly, cells were gated on FSC/SSC. Cell debris and dead cells were removed from the analysis based on size, complexity. Cells were gated on SSC-Height vs Area to discriminate doublets. Live cells were gated by the staining of Fixable Viability Dye eFluor 506. Cells incubated with IgG were used as negative control, setting the positive fluorescent signal captured as zero.

Fig. S9. *Irg1*-deficient macrophages acquire more pro-inflammatory features but less pro-angiogenic potential

(**A**) Single sample gene set enrichment analysis (ssGSEA) was conducted to calculate gene signature-based scores in intratumoral monocytes/macrophages in B16-F10 tumors. The enrichment score of indicated pathways between tumor-bearing *Irg1*$^{+/+}$ and *Irg1*$^{-/-}$ mice were compared, and the p values were calculated by an unpaired, two-tailed Student’s t test.

(**B** and **C**) BMDMs were challenged with B16-F10-TCM for 12 hours, followed by qRT-PCR to detect the mRNA expression (B) and protein expression (C) of indicated cytokines.

(**D** and **E**) BMDMs were co-cultured with B16-F10 (E) or stimulated with B16-F10-TCM (D) for 12 hours, followed by flow cytometry to determine the markers of M1/M2 polarization as indicated.

(**F**) BMDMs were challenged with B16-F10-TCM for 12 hours, followed by qRT-PCR to detect the mRNA expression of genes which were highly expressed in $Vegfa^+$ macrophages.

Data shown are the mean ± s.d. of four (B, D, E, and F) or eight (C) independent experiments. The p values were calculated by two-way ANOVA (B, D and E) and unpaired, two-tailed Student’s t test (C). $^{**}p < 0.01$, $^{****}p < 0.0001$ and n.s. denotes not significant.

Fig. S10. Irg1/Itaconate inhibits TET2 to regulate chemotaxis genes in TAMs

(**A and B**) 5hmC enrichment at the promoter regions of indicated genes were determined by hMeDIP-qPCR using TAMs isolated from B16-F10 melanoma (A) or BMDMs treated with or without B16-F10-TCM (B).

(**C**) 5hmC enrichment at the promoter regions of indicated genes were determined by hMeDIP-qPCR using BMDMs from $Tet2^{+/+}$ and $Tet2^{HxD}$ mice, as determined by qRT-PCR. The cells were pre-treated with 0.5 mM ITA for 12 hours, followed by treatment with B16-F10-TCM for another 12 hours.

(**D**) THP1 cells were differentiated to macrophages by treatment with Phorbol 12-myristate 13-acetate (PMA, 100 ng/mL) for 24 hours, and then were stimulated with MDA-MB-231-TCM for indicated time, following western-blot to detect IRG1 protein.

(**E**) *IRG1* was depleted in THP-1 cells by using sgRNA and was verified by western blot.

(**F**) 5hmC enrichment at the promoter regions of indicated genes were determined by hMeDIP-qPCR using THP-1 derived macrophages after treatment with or without MDA-MB-231-TCM for indicated time points, as determined by qRT-PCR. Data are the mean ± s.d. of four or three independent experiments. The p values were calculated by two-way ANOVA. *p < 0.05, **p < 0.01, ***p < 0.001, ****p < 0.0001 and n.s. denotes not significant.

Fig. S11. Irg1 does not affect the cytotoxicity of CD8$^+$ T cells in the TME

Flow cytometry analysis compared the cytotoxicity of T cells in B16-F10 tumors from *Irg1*$^{+/+}$ and *Irg1*$^{-/-}$ mice (n=8 per group). Percentages of cytotoxic CD8 T cells (Ifnγ$^+$Cd8$^+$), T cell cytotoxicity (Ifnγ$^+$ or Tnfα$^+$ of Cd8$^+$), exhausted T cells (Pd1$^+$ of Cd8$^+$) were measured as described in method. The p values were calculated by an unpaired, two-tailed Student's t test. **p < 0.01, and n.s. denotes not significant.

Fig. S12. *Irg1* deficiency in macrophages contributes to the tumor phenotype

(**A-B**) Flow cytometry analysis confirmed the proper depletion of TAMs (A) and TANs (B) in B16-F10 tumors from myeloid specific *Irg1*-KO mice and wild-type controls (n=6 per group).

(**C**) Experimental schematics of neutrophil and macrophage adoptive transfer assay (left). The purity of isolated neutrophils was confirmed by flow cytometry (right).

Fig. S13. KPC pancreatic tumors are resistant to anti-PD-1 immunotherapy

(**A**) KPC pancreatic cancer cells were inoculated orthotopically in the pancreas of *Irg1*$^{+/+}$ and *Irg1*$^{-/-}$ mice followed by i.p. injection of α-PD1 antibody or IgG control (n=5 per group). Tumors were harvested at day 19 post inoculation and tumor weight was measured. The p values were calculated by one-way ANOVA.

(**B**) The percentages of M1-like macrophages (Cd11b^{+}F4/80^{+} iNos^{+}) and CD8^{+} T cells (Cd3^{+}Cd8^{+}) in tumors as described in A were measured. The p values were calculated by one-way ANOVA (A) and unpaired, two-tailed Student's t test (B). *$p < 0.05$, **$p < 0.01$, and n.s. denotes not significant.

Fig. S14. Irg1 cannot affect the macrophage recruitment into KPC tumors

(**A** and **B**) Experimental schematics of labeled macrophage adoptive transfer. The recipients were i.v. injected with *Irg1*$^{+/+}$ or *Irg1*$^{-/-}$ BMDMs which were pre-labeled by FITC-conjugated beads (n=3 per group) at day 13, and tumors were harvested at day 14 post inoculation. KPC tumors were then subjected to flow cytometry analysis to detect the frequency of labeled TAMs (B). The p values were calculated by an unpaired, two-tailed Student's t test.

(**C**) IF staining of indicated markers in pancreatic tumors receiving macrophage therapy as described in Fig. 7C. Scale bar represents 20 μm. The quantification of average cell number was from 5 random high-power fields (HPFs). The p values were calculated by one-way ANOVA.

Fig. S15. Targeting *Irg1* in TAMs may reduce angiogenesis in KPC tumors

(**A**) Representative images of CD31 immunohistochemistry staining in KPC tumors receiving macrophage therapy as described in Fig. 5C. Scale bar represents 50 μm.

(**B** and **C**) IF staining of indicated markers in KPC tumors receiving macrophage therapy. Scale bar represents 50 μm. The quantification of average cell number from 5 random high-power fields (HPFs) is shown. *$p < 0.05$, **$p < 0.01$, ***$p < 0.001$ and n.s. denotes not significant.

Fig. S16. Irg1 acts as a master controller of macrophage antitumor activity

Tumor cells induce Irg1 expression in macrophages through NF-κB activation and subsequently lead to the accumulation of the immunomodulatory metabolite ITA, which dampens the inflammatory response in TAM subsets. As a result, Irg1/ITA alter the functional diversity of TAMs, such as restricted M1-like polarization and reduced pro-angiogenic potential in $Vegfa^+$ macrophages, and thereby creates and/or maintains the immunosuppressive TME favorable for tumor growth.

Fig. S1.
Positive correlation between *IRG1* mRNA expression and TAM fractions in multiple types of human tumors

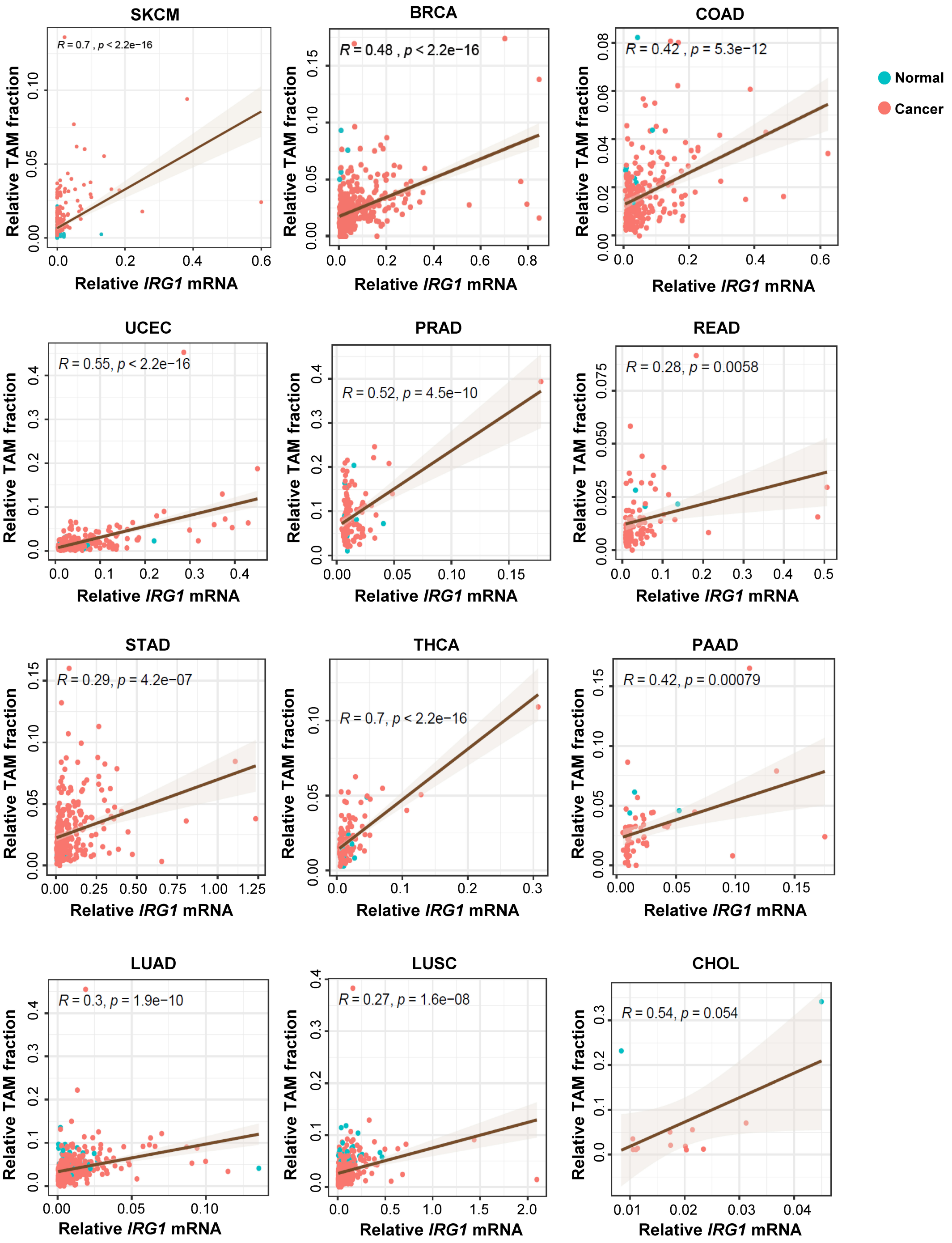

Fig. S2.
IRG1 is expressed in monocytes and macrophages in the TME of human cancer

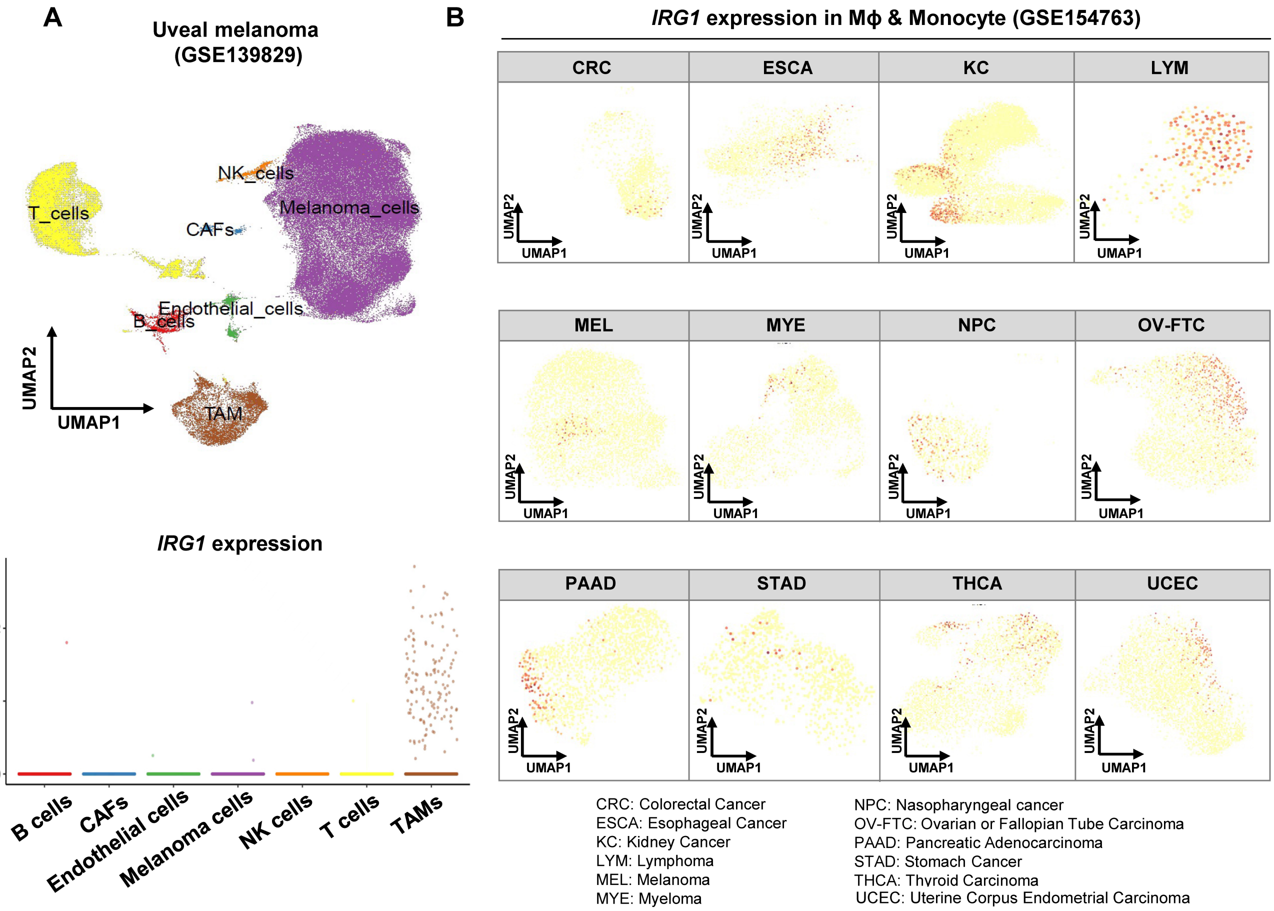

CRC: Colorectal Cancer
ESCA: Esophageal Cancer
KC: Kidney Cancer
LYM: Lymphoma
MEL: Melanoma
MYE: Myeloma
NPC: Nasopharyngeal cancer
OV-FTC: Ovarian or Fallopian Tube Carcinoma
PAAD: Pancreatic Adenocarcinoma
STAD: Stomach Cancer
THCA: Thyroid Carcinoma
UCEC: Uterine Corpus Endometrial Carcinoma

Fig. S3.
Tumor cells induce *Irg1* mRNA expression in macrophages through NF-κB activation

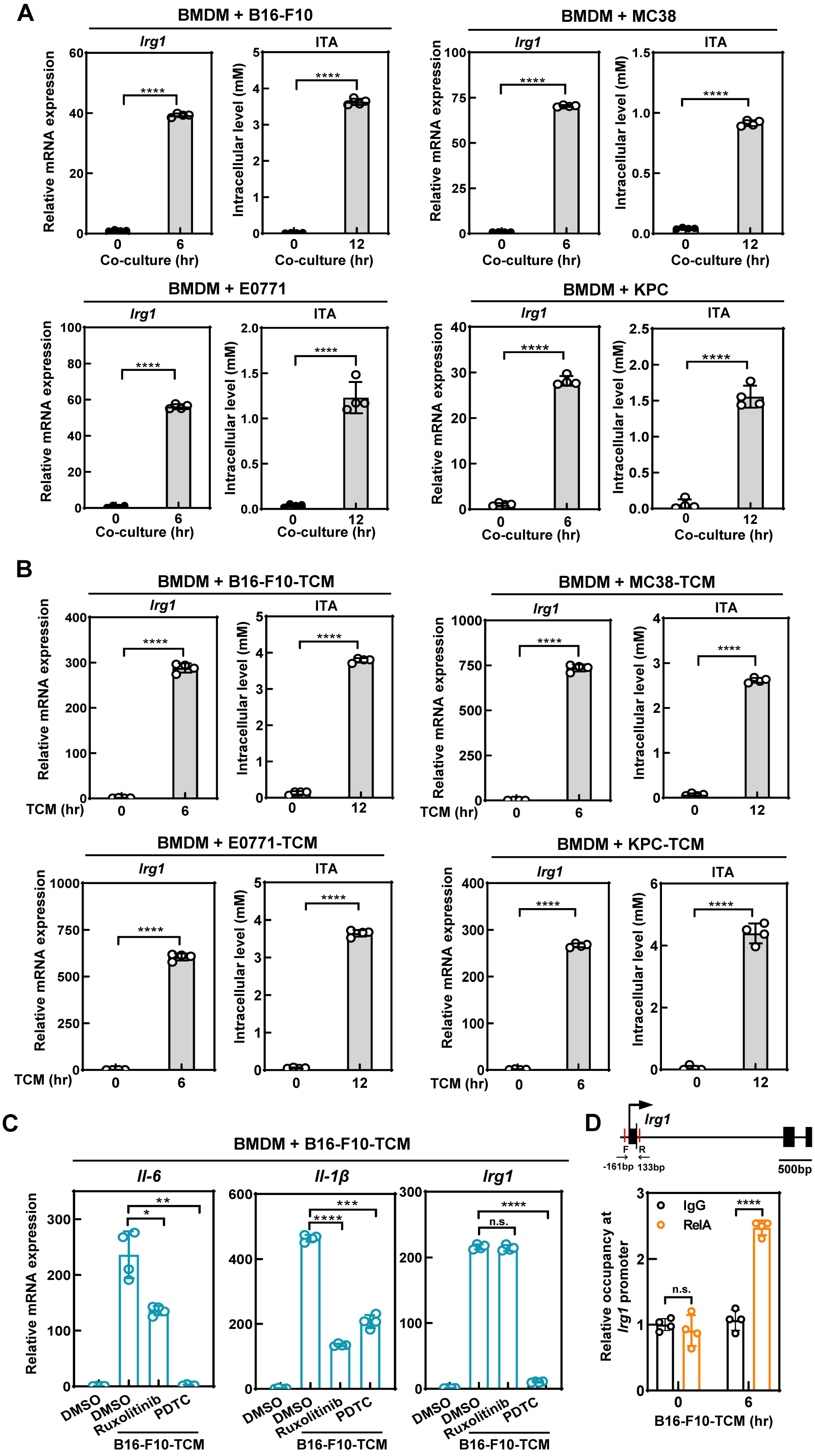

Fig. S4.
Irg1 deficiency inhibits tumor growth in immune competent mice

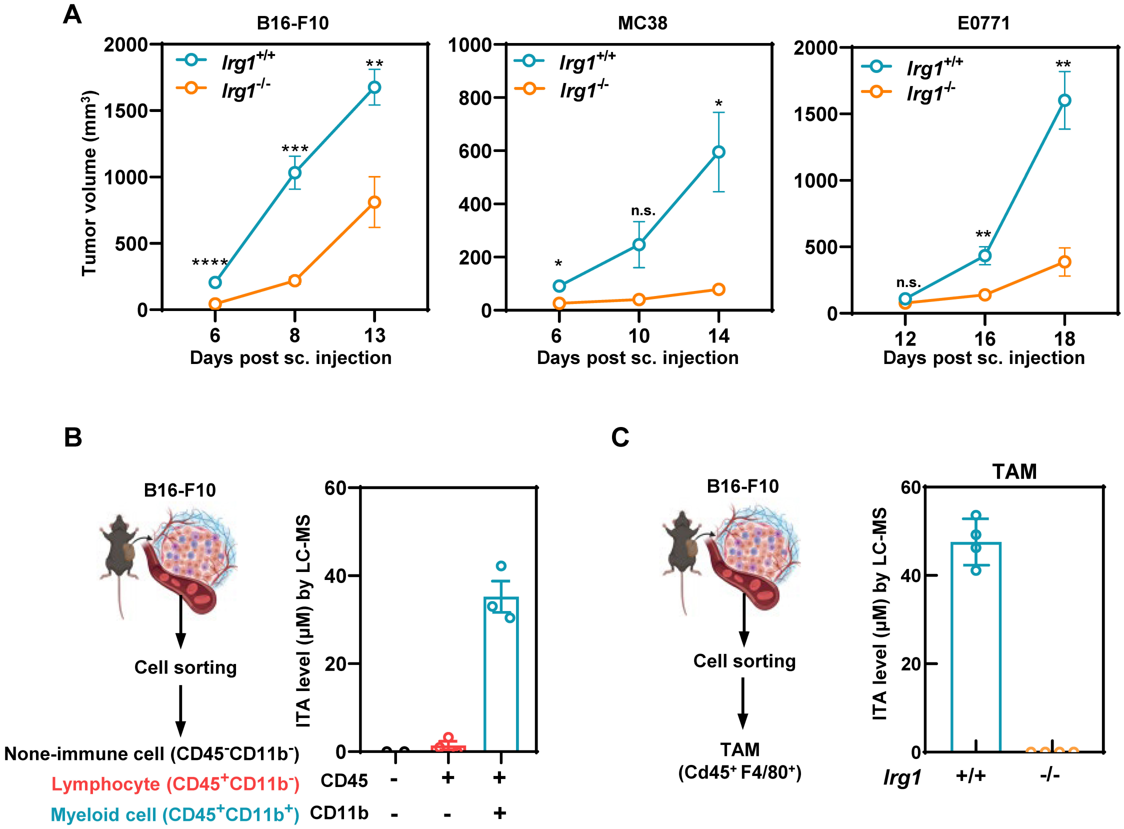

Fig. S5.
Myeloid-specific *Irg1* deficiency inhibits tumor growth in immune competent mice

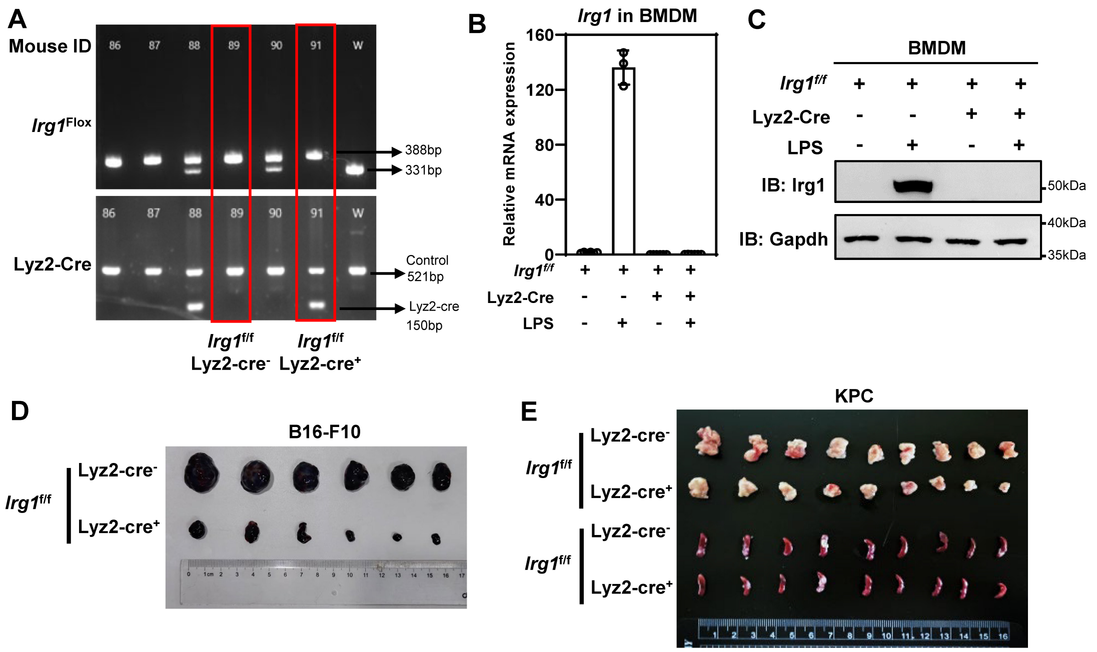

Fig. S6.
scRNA-seq unveils major immune populations in the TME of B16-F10 tumor

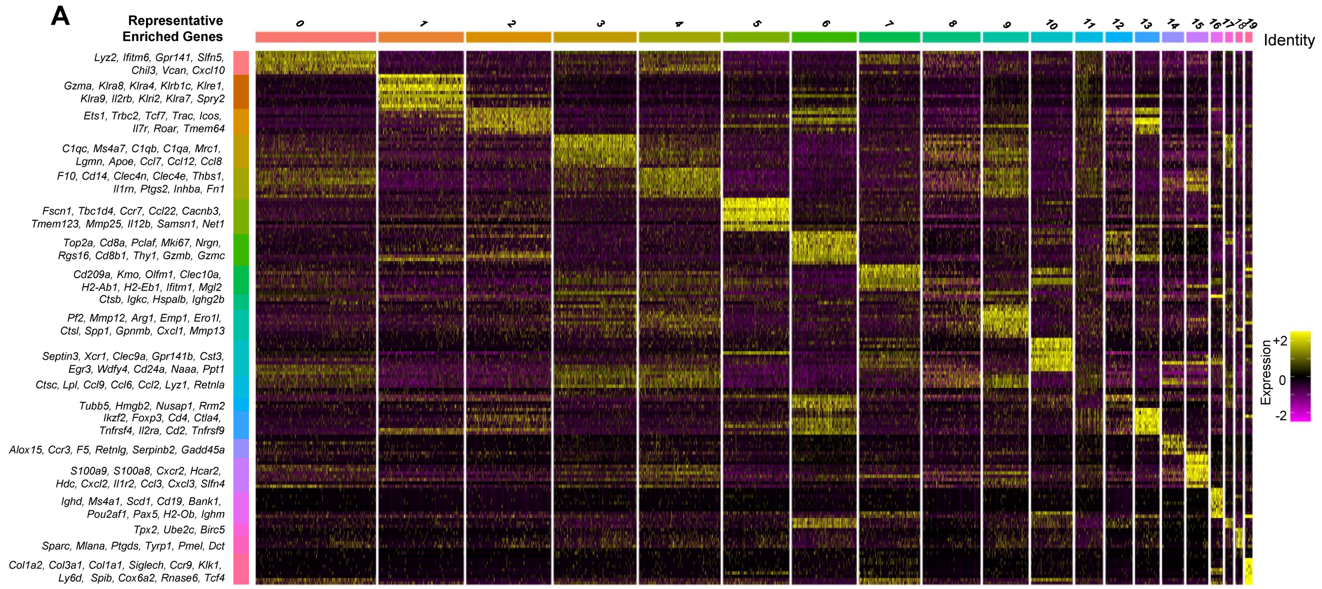

B

Cluster	Cell type	Marker
0	Mϕ & Monocyte	*Csf1r,C1qa,Csf3r,Vcan*
1	NK	*Ncr1,Klrb1c*
2	T	*Cd3d,Cd3e*
3	Mϕ & Monocyte	*Csf1r,C1qa,Csf3r,Vcan*
4	Mϕ & Monocyte	*Csf1r,C1qa,Csf3r,Vcan*
5	DC	*Flt3,Fscn1*
6	T	*Cd3d,Cd3e*
7	DC	*Flt3,Cd209a*
8	Mϕ & Monocyte	*Csf1r,C1qa,Csf3r,Vcan*
9	Mϕ & Monocyte	*Csf1r,C1qa,Csf3r,Vcan*
10	DC	*Flt3,Xcr1*
11	Mϕ & Monocyte	*Csf1r,C1qa,Csf3r,Vcan*
12	T	*Cd3d,Cd3e*
13	T	*Cd3d,Cd3e*
14	Neutrophil	*Retnlg,G0s2,Ccr3*
15	Neutrophil	*Retnlg,G0s2,S100a9*
16	B	*Cd79a,Ms4a1,Cd19*
17	Mϕ & Monocyte	*Csf1r,C1qa,Csf3r,Vcan*
18	Melanocyte	*Tyrp1,Dct*
19	pDC	*Siglech,Ccr9*

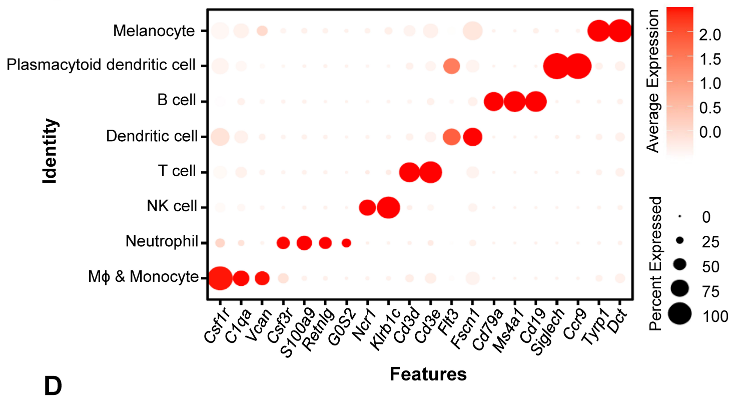

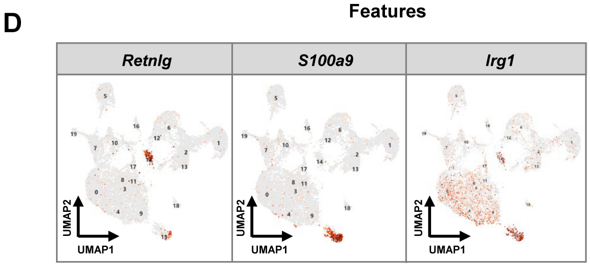

Fig. S7.
Developmental trajectory of monocytes and macrophage subsets in the TME of B16-F10 tumor

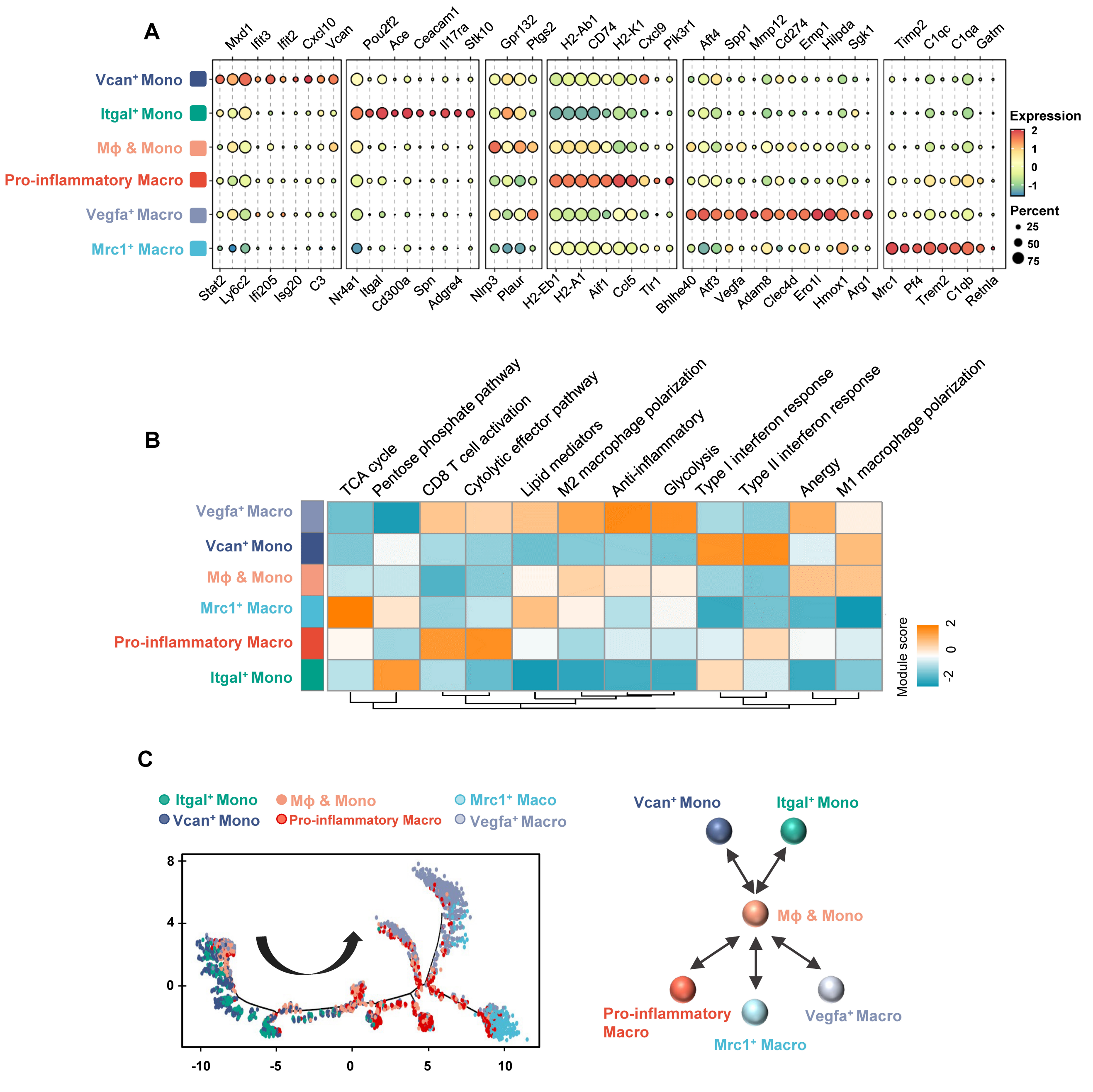

Fig. S8.
The gating strategy for flow cytometry data

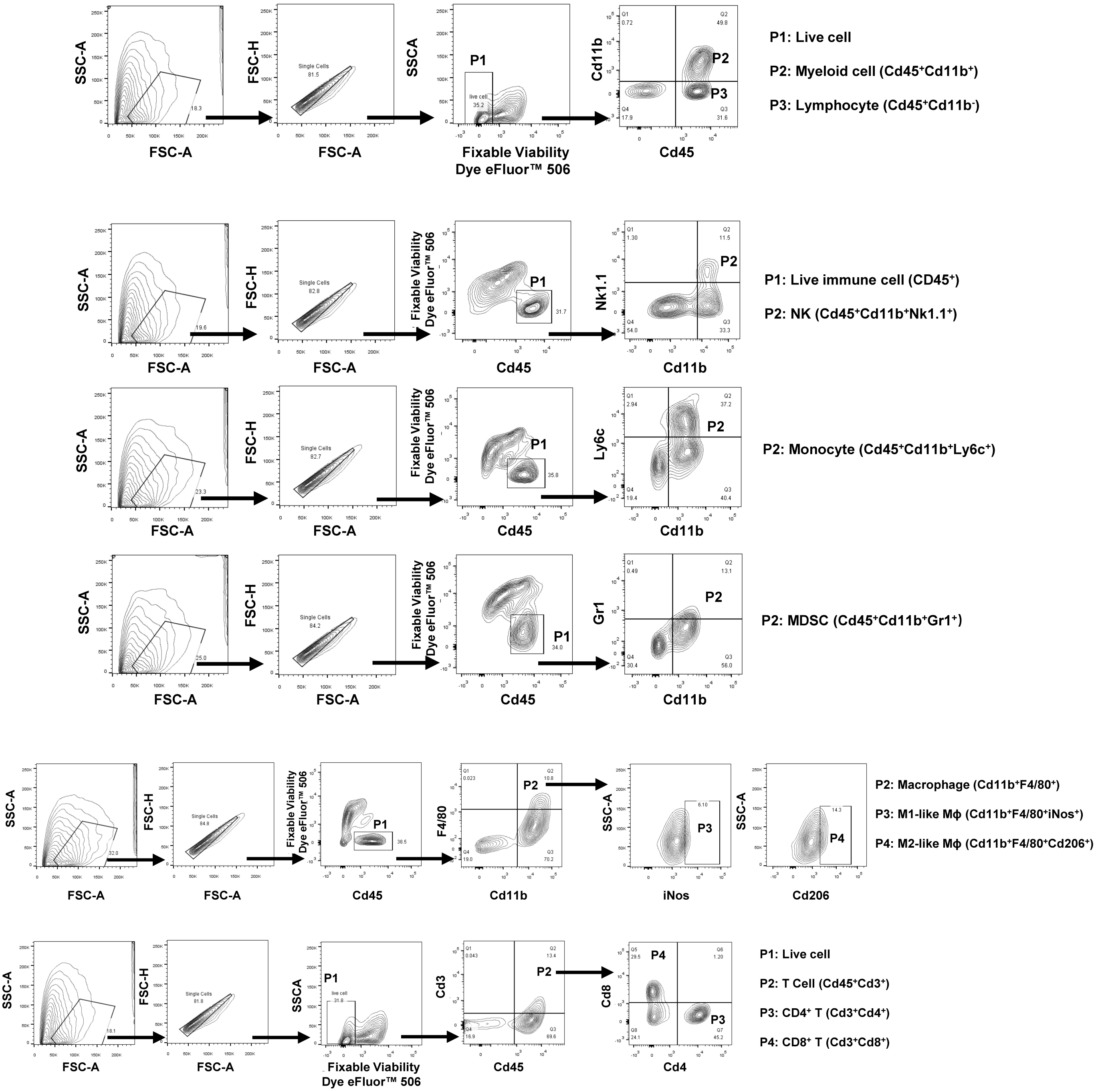

Fig. S9.
Irg1-deficient macrophages acquire more pro-inflammatory features but less pro-angiogenic potential

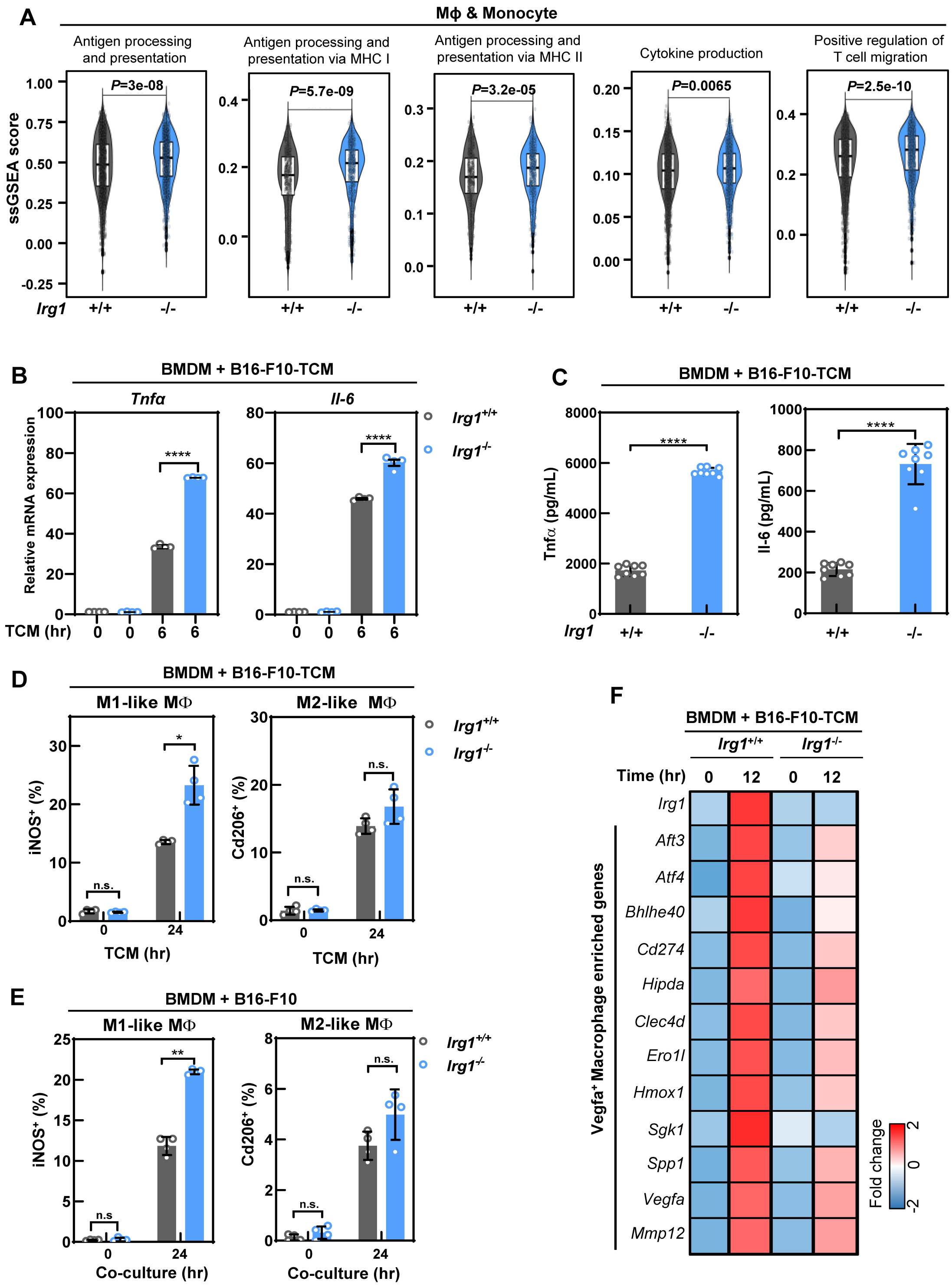

Fig. S10.
Irg1/Itaconate inhibits Tet2 to regulate chemotaxis genes in TAMs

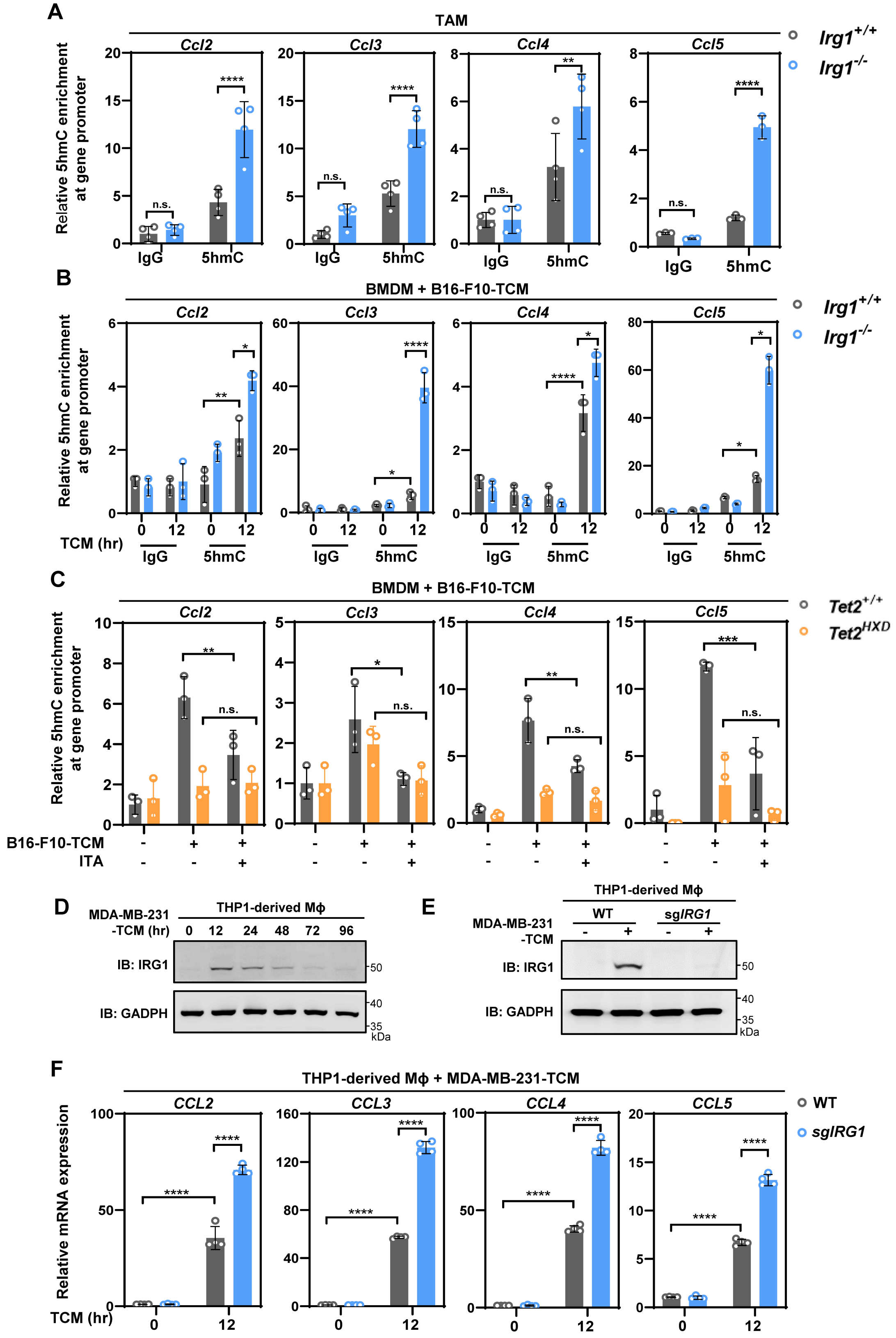

Fig. S11.
Irg1 may not directly affect the cytotoxicity of CD8⁺ T cells

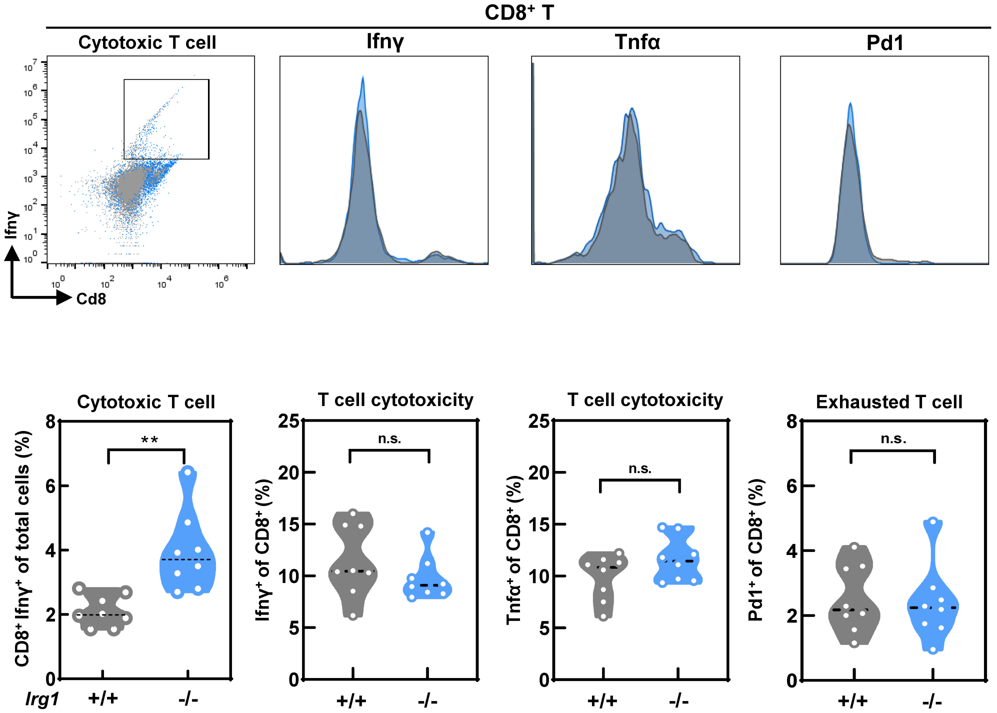

Fig. S12.

Irg1 deficiency in macrophages contributes to the tumor phenotype

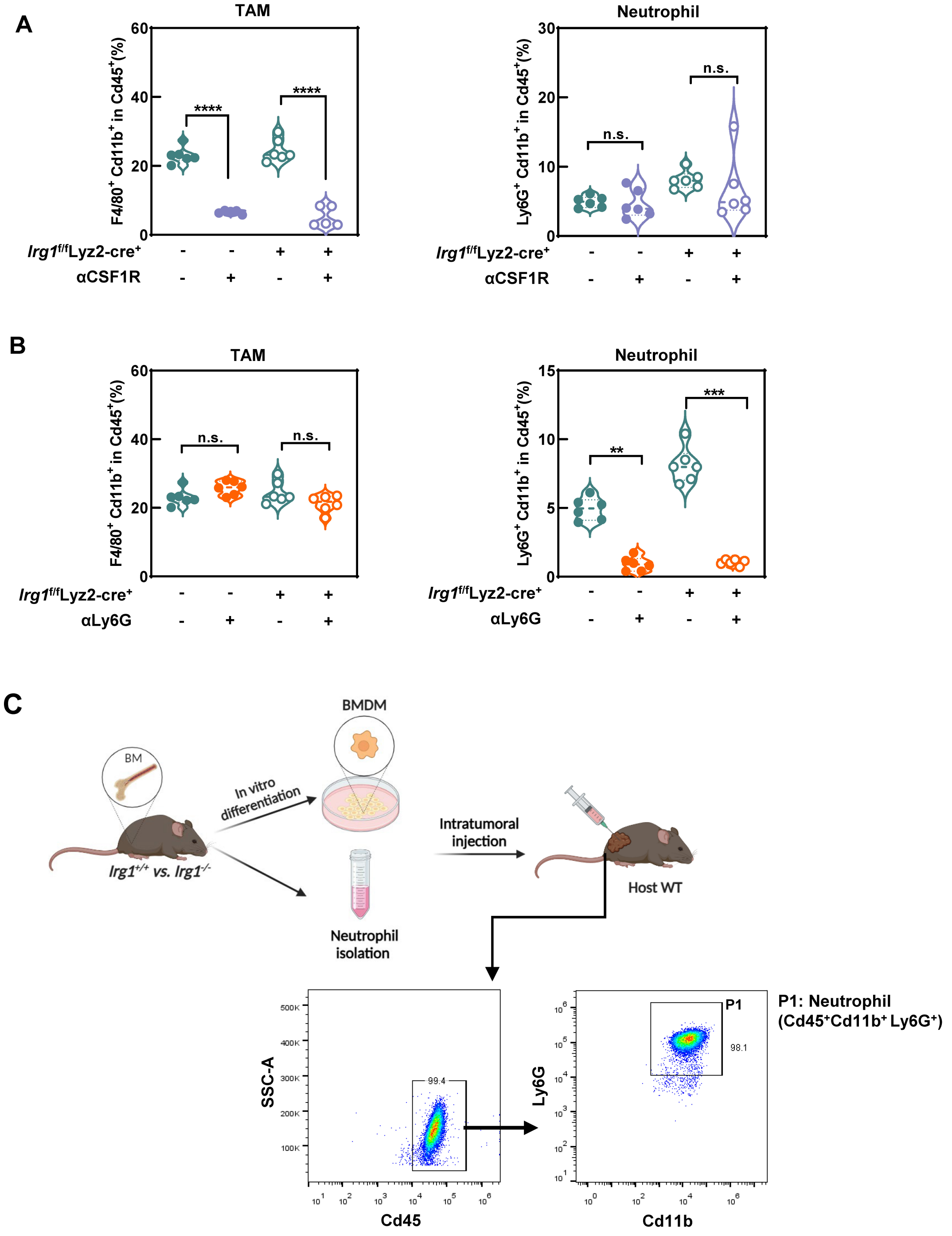

Fig. S13.
KPC pancreatic tumors are resistant to anti-PD1 immunotherapy

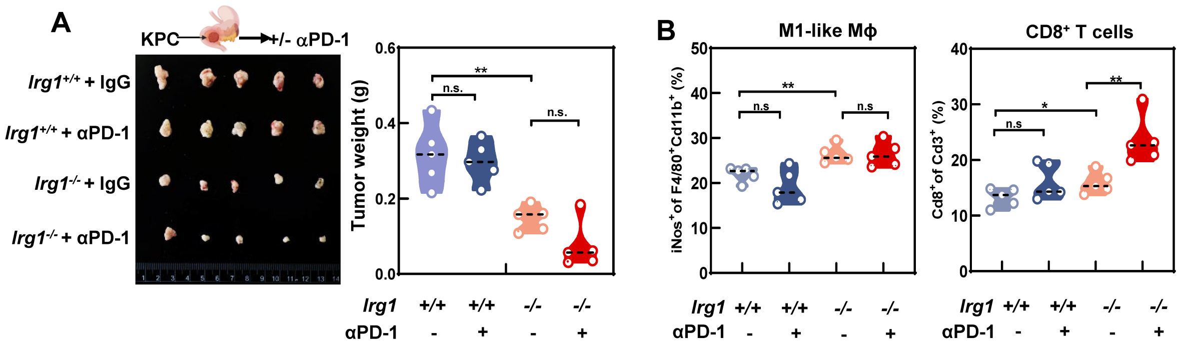

Fig. S14.
Irg1 cannot affect the macrophage recruitment into KPC tumors

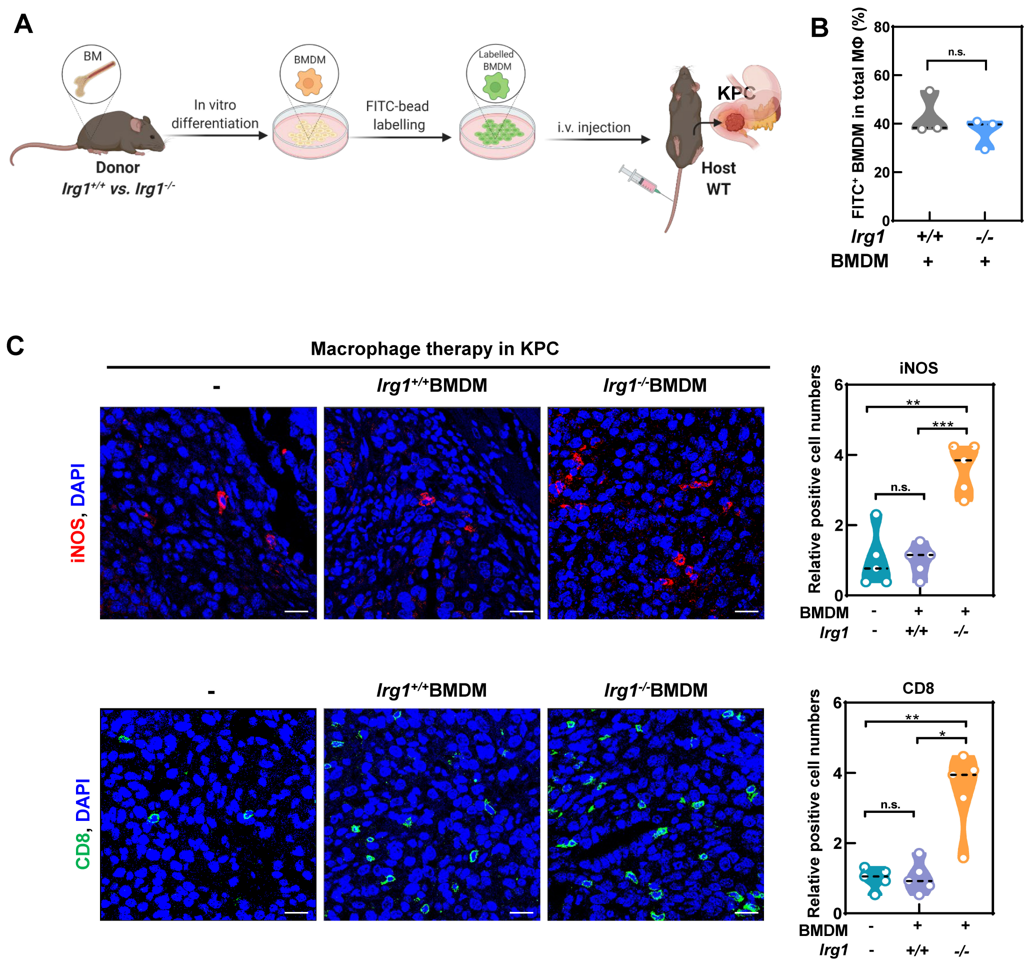

Fig. S15.
Targeting Irg1 in TAMs may reduce angiogenesis in KPC tumors

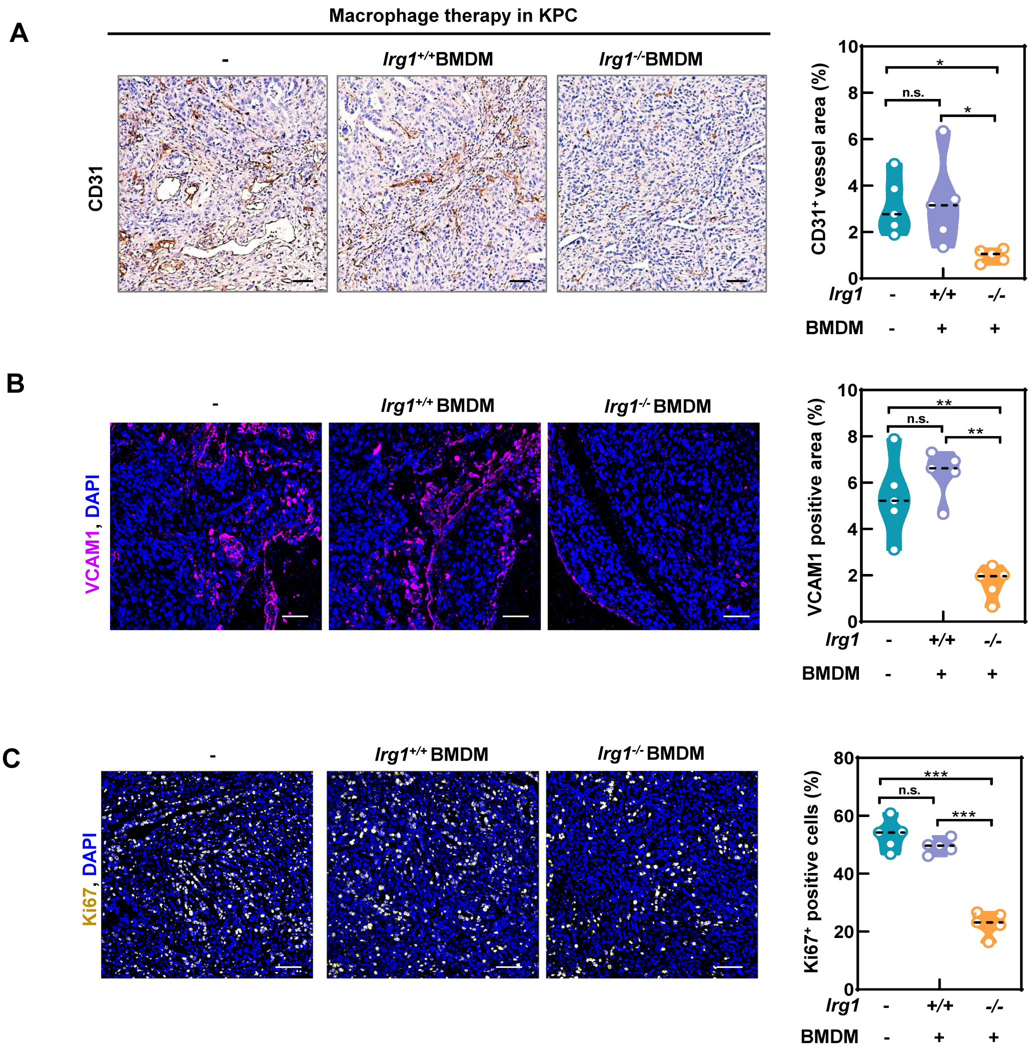

Fig. S16.
Irg1 acts as a master controller of macrophage antitumor activity

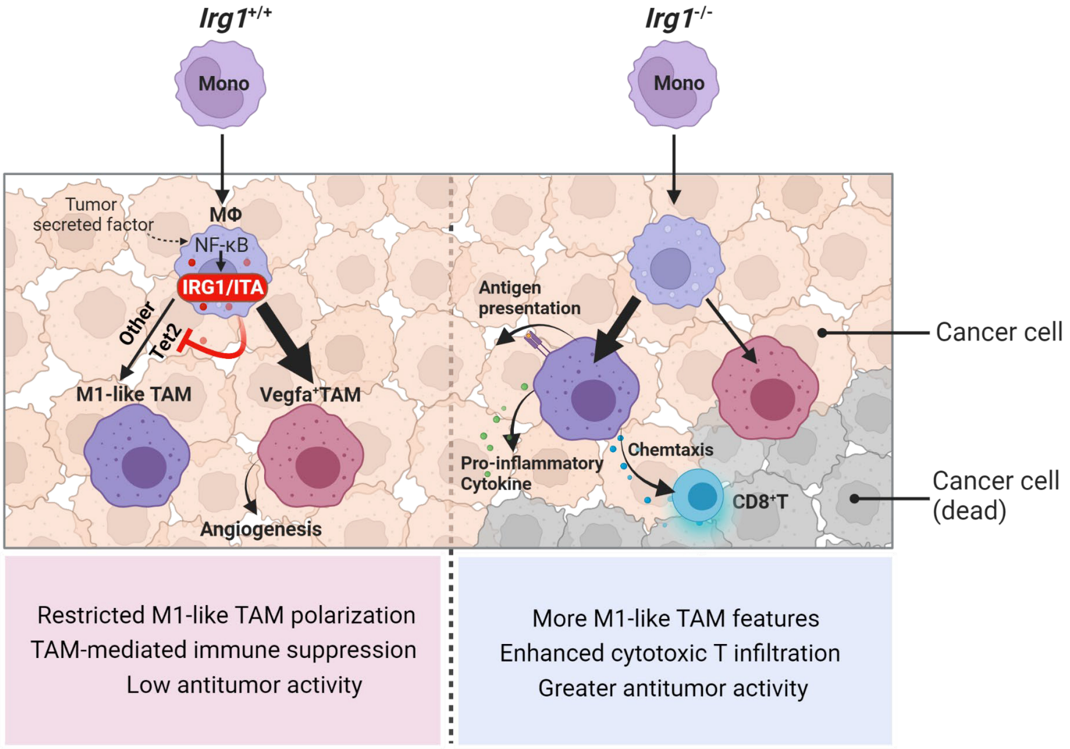

Table S1. The general information of the scRNA-seq

Sample	Irg1$^{+/+}$	Irg1$^{-/-}$
Estimated Number of Cells	6408	5896
Fraction Reads in Cells	0.8909	0.9026
Median Genes per Cell	1993	1971
Total Genes Detected	19050	18601
Median UMI Counts per Cell	4560	4635
Confidently Mapping Reads per Cell	23007.29	24449.64
Reads Mapped Confidently to Genome	0.8548	0.8512
Reads Mapped Confidently to Intergenic Regions	0.0318	0.0362
Reads Mapped Confidently to Intronic Regions	0.0704	0.0704
Reads Mapped Confidently to Exonic Regions	0.7526	0.7446
Sequencing Saturation	0.6438	0.6727
MappedReadsPerCells	23007.29	24449.64
FranctionKept	1	0.941

Table S2. Clinical characteristics of the 34 HCC patients

Number	Origin	Treatment response	Age	Gender		Liver cancer staging *	Liver cirrhosis	Tumor diameter, cm	Lymphoid metastasis	MVI	Differentiation	HBsAg	AFP, ng/mL		ALT, U/L	AST, U/L	GGT, U/L
				Male	Female								＜20	≥20			
133	Zhongshan hospital	√	52	√		Iia	×	3.5*3*2; 1.1	×	√	II	√		√	65	67	43
136	Zhongshan hospital	×	58	√		Ia	×	1.1	×	×	II	√		√	44	68	55
139	Zhongshan hospital	×	69	√		Ia	×	1.1	×	×	II	√		√	78	56	46
144	Zhongshan hospital	×	44		√	Ia	×	3.8*3.5*3, 2*1.5*1.5	×	×	II	√		√	88	89	56
147	Zhongshan hospital	×	51	√		Ia	×	2*1.6*1.4	×	×	II	√		√	89	99	78
148	Zhongshan hospital	√	68	√		Ib	√	2.3; 1	×	√	III	×		√	89	76	65
149	Zhongshan hospital	×	48	√		Ib	×	1; 1	×	√	II	√		√	77	79	45
150	Zhongshan hospital	×	62	√		Ia	×	3*2.5*2	×	√	I	√	√		78	87	43
151	Zhongshan hospital	×	71		√	Ib	×	2.5*2.5*2, 1.5*1.5*0.8	×	×	II	√	√		98	88	67
153	Zhongshan hospital	×	66	√		Ia	×	1	×	×	I	√		√	68	89	65
154	Zhongshan hospital	×	68	√		Ia	√	1.3	×	×	II	×		√	99	76	76
155	Zhongshan hospital	√	52	√		Ia	×	2	×	×	II	√		√	66	69	74
156	Zhongshan hospital	√	73	√		Ib	×	6*5.5*4.7	×	×	II	×		√	88	77	43
157	Zhong shan hospital	√	58		√	Ia	×	3.5*3*2.5	×	×	II	√		√	86	79	56
	Zhongshan hospital	√	58			Ia	×	3.5*3*2.5	×	×	II	√		√	86	79	56
	Zhong shan hospital	√	65			Ib	√	5*4*3	×	×	III	√		√	42	44	34
D19-00701	Huashan hospital	√	58	√		C	√	12	√	NE	NE	√		√	87	182	230
D19-01658	Huashan hospital	√	42	√		B	√	8*3	×	√	II	√		√	368	132	40
D19-01888	Huashan hospital	×	72		√	B	√	7.5	×	×	II	×		√	227	290	705
D19-02171	Huashan hospital	√	65	√		C	√	8	×	√	II~III	√		√	42	45	204
D19-02434	Huashan hospital	×	48	√		B	√	10	×	√	II	√	√		24	42	156
D19-02922	Huashan hospital	√	59	√		C	×	4	×	√	II	√		√	404	312	89
D19-03153	Huashan hospital	√	45	√		B	×	12	×	√	II	√		√	59	128	97
D20-01092	Huashan hospital	√	53	√		B	×	5.5	×	√	III	×		√	24	20	27
D20-01959	Huashan hospital	√	55	√		B	×	8.5	×	√	III	√		√	83	97	172
D20-02757	Huashan hospital	√	62		√	B	√	4*0.8	×	√	II~III	√		√	28	47	65
D21-00203	Huashan hospital	×	43	√		C	√	14	×	√	III	√		√	24	47	133
D21-00389	Huashan hospital	√	48	√		B	√	6	×	√	III	√	√		26	24	57
D21-00523	Huashan hospital	√	69	√		B	√	9	×	√	II	√	√		51	42	53
D21-01027	Huashan hospital	√	58	√		C	√	12	√	NE	NE	√		√	87	182	230
D21-02363	Huashan hospital	√	44	√		C	√	7.8	×	√	II	√	√		36	24	117
D22-00128	Huashan hospital	√	75		√	C	√	6.5	×	NE	NE	√		√	66	86	577
D22-01461	Huashan hospital	√	72	√		B	×	17	×	NE	I	×		√	29	19	119
D20-00806	Huashan hospital	√	61	√		B	√	6*3	×	√	NE	√	√		42	58	61

*BCLC stage: Zhongshan hospital evaluate the patients by China liver cancer staging. Huashan hospital evaluate the patients by Barcelona Clinic Liver Cancer staging.

NE: Not evaluated

MVI: Microvascular invasion

HBsAg: Hepatitis B surface antigen

AFP: alpha-fetoprotein

ALT: Alanine Transaminase

Table S3. Sequences of primers used in this study

Gene	Sequence	Application	Species
Irg1-F	AGTTTTCTGGCCTCGACCTG	qRT-PCR	MUS
Irg1-R	AGAGGGAGGGTGGAATCTCT	qRT-PCR	MUS
Il-1β-F	CAACCAACACGTGATATTCTCCATG	qRT-PCR	MUS
Il-1β-R	GATCCACACTCTCCAGCTGCA	qRT-PCR	MUS
Il6-F	TCCATCCAGTTGCCTTCTTG	qRT-PCR	MUS
Il6-R	TTCCACGATTTCCCAGAGAA	qRT-PCR	MUS
Tnfα-F	ACCCACGGCTCCACCCTCTC	qRT-PCR	MUS
Tnfα-R	CCCTCTGGGGGCCGATCACT	qRT-PCR	MUS
Cd206-F	CTCTGTTCAGCTATTGGACGC	qRT-PCR	MUS
Cd206-R	CGGAATTTCTGGGATTCAGCTTC	qRT-PCR	MUS
Cd80-F	ACCCCCAACATAACTGAGTCT	qRT-PCR	MUS
Cd80-R	TTCCAACCAAGAGAAGCGAGG	qRT-PCR	MUS
Cd86-F	TGTTTCCGTGGAGACGCAAG	qRT-PCR	MUS
Cd86-R	TTGAGCCTTTGTAAATGGGCA	qRT-PCR	MUS
Rn18S-F	CGCGGTTCTATTTTGTTGGT	qRT-PCR	MUS
Rn18S-R	AGTCGGCATCGTTTATGGTC	qRT-PCR	MUS
Arg1-F	CTCCAAGCCAAAGTCCTTAGAG	qRT-PCR	MUS
Arg1-R	AGGAGCTGTCATTAGGGACATC	qRT-PCR	MUS
Ym1-F	AGAAGGGAGTTTCAAACCTGGT	qRT-PCR	MUS
Ym1-R	GTCTTGCTCATGTGTGTAAGTGA	qRT-PCR	MUS
Hif1α-F	ACCTTCATCGGAAACTCCAAAG	qRT-PCR	MUS
Hif1α-R	CTGTTAGGCTGGGAAAAGTTAGG	qRT-PCR	MUS
iNos-F	GTTCTCAGCCCAACAATACAAGA	qRT-PCR	MUS
iNos-R	GTGGACGGGTCGATGTCAC	qRT-PCR	MUS
Ccl2-F	TAAAAACCTGGATCGGAACCAAA	qRT-PCR	MUS
Ccl2-R	GCATTAGCTTCAGATTTACGGGT	qRT-PCR	MUS
Ccl3-F	TTCTCTGTACCATGACACTCTGC	qRT-PCR	MUS
Ccl3-R	CGTGGAATCTTCCGGCTGTAG	qRT-PCR	MUS
Ccl4-F	TTCCTGCTGTTTCTCTTACACCT	qRT-PCR	MUS
Ccl4-R	CTGTCTGCCTCTTTTGGTCAG	qRT-PCR	MUS
Ccl5-F	GCTGCTTTGCCTACCTCTCC	qRT-PCR	MUS
Ccl5-R	TCGAGTGACAAACACGACTGC	qRT-PCR	MUS
Cxcl9-F	GAGCAGTGTGGAGTTCGAGG	qRT-PCR	MUS
Cxcl9-R	TCCGGATCTAGGCAGGTTTG	qRT-PCR	MUS
Cxcl10-F	AATGAGGGCCATAGGGAAGC	qRT-PCR	MUS
Cxcl10-R	AGCCATCCACTGGGTAAAGG	qRT-PCR	MUS
Ox40-F	TACCTACCCCAGTGGTCACAA	qRT-PCR	MUS
Ox40-R	ACGGATGACATAGAGTATCCCTG	qRT-PCR	MUS
Icosl-F	TAAAGTGTCCCTGTTTTGTGTCC	qRT-PCR	MUS
Icosl-R	ATTGCACCGACTTCAGTCTCT	qRT-PCR	MUS

Cd40-F	TGTCATCTGTGAAAAGGTGGTC	qRT-PCR	MUS
Cd40-R	ACTGGAGCAGCGGTGTTATG	qRT-PCR	MUS
Icam1-F	GTGATGCTCAGGTATCCATCCA	qRT-PCR	MUS
Icam1-R	CACAGTTCTCAAAGCACAGCG	qRT-PCR	MUS
Ifna2-F	TACTCAGCAGACCTTGAACCT	qRT-PCR	MUS
Ifna2-R	CAGTCTTGGCAGCAAGTTGAC	qRT-PCR	MUS
Ifnb1-F	CAGCTCCAAGAAAGGACGAAC	qRT-PCR	MUS
Ifnb1-R	GGCAGTGTAACTCTTCTGCAT	qRT-PCR	MUS
Il27-F	CTGTTGCTGCTACCCTTGCTT	qRT-PCR	MUS
Il27-R	CACTCCTGGCAATCGAGATTC	qRT-PCR	MUS
Il23a-F	ATGCTGGATTGCAGAGCAGTA	qRT-PCR	MUS
Il23a-R	ACGGGGCACATTATTTTTAGTCT	qRT-PCR	MUS
Atf3-F	GAGGATTTTGCTAACCTGACACC	qRT-PCR	MUS
Atf3-R	TTGACGGTAACTGACTCCAGC	qRT-PCR	MUS
Atf4-F	ATGGCGCTCTTCACGAAATC	qRT-PCR	MUS
Atf4-R	ACTGGTCGAAGGGGTCATCAA	qRT-PCR	MUS
Bhlhe40-F	ACGGAGACCTGTCAGGGATG	qRT-PCR	MUS
Bhlhe40-R	GGCAGTTTGTAAGTTTCCTTGC	qRT-PCR	MUS
Cd274-F	GCTCCAAAGGACTTGTACGTG	qRT-PCR	MUS
Cd274-R	TGATCTGAAGGGCAGCATTTC	qRT-PCR	MUS
Hilpda -F	TGCTGGGCATCATGTTGACC	qRT-PCR	MUS
Hilpda -R	TGACCCCTCGTGATCCAGG	qRT-PCR	MUS
Clec4d-F	ACCCGACATCCCCAACTGAT	qRT-PCR	MUS
Clec4d-R	CTCTCGTCCAGCGTAAAAAGT	qRT-PCR	MUS
Ero1l-F	TTCTGCCAGGTTAGTGGTTACC	qRT-PCR	MUS
Ero1l-R	GTTTGACGGCACAGTCTCTTC	qRT-PCR	MUS
Hmox1-F	AAGCCGAGAATGCTGAGTTCA	qRT-PCR	MUS
Hmox1-R	GCCGTGTAGATATGGTACAAGGA	qRT-PCR	MUS
Sgk1 -F	CTGCTCGAAGCACCCTTACC	qRT-PCR	MUS
Sgk1 -R	TCCTGAGGATGGGACATTTTCA	qRT-PCR	MUS
Spp1-F	AGCAAGAAACTCTTCCAAGCAA	qRT-PCR	MUS
Spp1-R	GTGAGATTCGTCAGATTCATCCG	qRT-PCR	MUS
Vegfa-F	GCACATAGAGAGAATGAGCTTCC	qRT-PCR	MUS
Vegfa-R	CTCCGCTCTGAACAAGGCT	qRT-PCR	MUS
Mmp12 -F	GAGTCCAGCCACCAACATTAC	qRT-PCR	MUS
Mmp12 -R	GCGAAGTGGGTCAAAGACAG	qRT-PCR	MUS
Ccl2-F	TGGAAACACCCGAGGGCTCT	hMeDIP–qPCR	MUS
Ccl2-R	CCTTCCTCCCGTCTGGCTCT	hMeDIP–qPCR	MUS
Ccl3-F	AGTCTCAGCTCTCAACTCGT	hMeDIP–qPCR	MUS
Ccl3-R	CACAGCTATTCTTCCATGTGGT	hMeDIP–qPCR	MUS
Ccl4-F	GGTTCACAACCCCCCTTCTCT	hMeDIP–qPCR	MUS
Ccl4-R	GGTTGCAGGAAGGAAAACGGAAT	hMeDIP–qPCR	MUS
Ccl5-F	ACTTACATGGTGAGGCAGGT	hMeDIP–qPCR	MUS

Ccl5-R	GTGGAAACTCCCCAAGTCCT	hMeDIP–qPCR	MUS
Il6-F	CATCAAGACATGCTCAAGTGCT	hMeDIP–qPCR	MUS
Il6-R	ACTCATGGGAAAATCCCACAT	hMeDIP–qPCR	MUS
Il-1β-F	CAGGGTTTGTTGTCCAACTTGT	hMeDIP–qPCR	MUS
Il-1β-R	CGATGACACACTTGCGAATGT	hMeDIP–qPCR	MUS
Cxcl9-F	GAGGAAAAGAACAGCGGACT	hMeDIP–qPCR	MUS
Cxcl9-R	CCAGGACGATCAATTTGTGGT	hMeDIP–qPCR	MUS
Cxcl10-F	CGGAAGGTTGGCTCGGGAT	hMeDIP–qPCR	MUS
Cxcl10-R	CACAATAAACACAAGCAATGCCCT	hMeDIP–qPCR	MUS
Irg1-F	AATGGGCTGTCTGTGAGA	ChIP-qPCR	MUS
Irg1-R	CCAGGTCTGTCCCTTTCAC	ChIP-qPCR	MUS
ACOD1-F	TGGGGCCTTTTATGCCAACT	qRT-PCR	HOMO
ACOD1-R	CTCACCTGTGGCCTGTTGAT	qRT-PCR	HOMO
ACTIN-F	GCCGACAGGATGCAGAAGGAGATCA	qRT-PCR	HOMO
ACTIN-R	AAGCATTTGCGGTGGACGATGGA	qRT-PCR	HOMO
CCL2-F	CAGCCAGATGCAATCAATGCC	qRT-PCR	HOMO
CCL2-R	TGGAATCCTGAACCCACTTCT	qRT-PCR	HOMO
CCL3-F	AGTTCTCTGCATCACTTGCTG	qRT-PCR	HOMO
CCL3-R	CGGCTTCGCTTGGTTAGGAA	qRT-PCR	HOMO
CCL4-F	CTGTGCTGATCCCAGTGAATC	qRT-PCR	HOMO
CCL4-R	TCAGTTCAGTTCCAGGTCATACA	qRT-PCR	HOMO
CCL5-F	CCAGCAGTCGTCTTTGTCAC	qRT-PCR	HOMO
CCL5-R	CTCTGGGTTGGCACACACTT	qRT-PCR	HOMO
CXCL9-F	CCAGTAGTGAGAAAGGGTCGC	qRT-PCR	HOMO
CXCL9-R	AGGGCTTGGGGCAAATTGTT	qRT-PCR	HOMO
CXCL10-F	GTGGCATTCAAGGAGTACCTC	qRT-PCR	HOMO
CXCL10-R	TGATGGCCTTCGATTCTGGATT	qRT-PCR	HOMO
